# Berberine attenuates uric acid-induced cell injury by inhibiting NLRP3 signaling pathway in HK-2 cells

**DOI:** 10.1007/s00210-023-02451-3

**Published:** 2023-05-17

**Authors:** Jingna Zheng, Shiting Gong, Gong Wu, Xiaohong Zheng, Jincan Li, Juan Nie, Yanlu Liu, Baoyi Chen, Yuhong Liu, Ziren Su, Jiannan Chen, Yucui Li

**Affiliations:** 1 School of Pharmaceutical Sciences, Guangzhou University of Chinese Medicine, Guangzhou Higher Education Mega Center, 232# Wai Huan East Road, Guangzhou, 510006 Guangdong China; 2 Department of TCM Orthopedics & Traumatology, Orthopedic Hospital of Longgang, Shenzhen, 518116 Guangdong China; 3 Medical School, Hubei Minzu University, Enshi, 445000 Hubei China; 4 School of Food and Pharmaceutical Engineering, Zhaoqing University, Zhaoqing, 526040 Guangdong China; 5 Dongguan Institute of Guangzhou University of Chinese Medicine, Dongguan, 523808 Guangdong China

**Keywords:** Hyperuricemia, Berberine, NLRP3, Uric acid

## Abstract

Hyperuricemia (HUA) is a common chronic metabolic disease that can cause renal failure and even death in severe cases. Berberine (BBR) is an isoquinoline alkaloid derived from *Phellodendri Cortex* with strong antioxidant, anti-inflammatory, and anti-apoptotic properties. The purpose of this study was to investigate the protective effects of berberine (BBR) against uric acid (UA)-induced HK-2 cells and unravel their regulatory potential mechanisms. The CCK8 assay was carried out to detect cell viability. The expression levels of inflammatory factors interleukin-1β (IL-1β) and interleukin-18 (IL-18) and Lactate dehydrogenase (LDH) were measured using Enzyme-linked immunosorbent assays (ELISA). The expression of the apoptosis-related protein (cleaved-Caspase3, cleaved-Caspase9, BAX, BCL-2) was detected by western blot. The effects of BBR on the activities of the NOD-like receptor family pyrin domain containing 3 (NLRP3) and the expression of the downstream genes were determined by RT-PCR and western blot in HK-2 cells. From the data, BBR significantly reversed the up-regulation of inflammatory factors (IL-1β, IL-18) and LDH. Furthermore, BBR down-regulated protein expression of pro-apoptotic proteins BAX, cleaved caspase3 (cl-Caspase3), cleaved caspase9 (cl-Caspase9), and enhanced the expression of antiapoptotic protein BCL-2. Simultaneously, BBR inhibited the activated NLPR3 and reduced the mRNA levels of NLRP3, Caspase1, IL-18, and IL-1β. Also, BBR attenuated the expression of NLRP3 pathway-related proteins (NLRP3, ASC, Caspase1, cleaved-Caspase1, IL-18, IL-1β, and GSDMD). Furthermore, specific NLRP3-siRNA efficiently blocked UA-induced the level of inflammatory factors (IL-1β, IL-18) and LDH and further inhibited activated NLRP3 pathway. Collectively, our results suggested that BBR can alleviate cell injury induced by UA. The underlying unctionary mechanism may be through the NLRP3 signaling pathway.

**Supplementary Information:**

The online version contains supplementary material available at 10.1007/s00210-023-02451-3.

## Introduction

Hyperuricemia (HUA) is a chronic metabolic disease, often characterized by abnormally elevated levels of serum uric acid (UA) because of purine metabolism disorder or abnormal urine metabolism, slowly evolving towards renal fibrosis and renal failure (Grayson et al. 2011). In severe cases, it may result in various complications and even lead to gout and death, posing a great threat to human health (Hyperuricemia 2015; Dong et al. 2017). Previous research illustrated that modern high-protein, high-purine diets, and irregular lifestyles contributed to a greater prevalence of HUA and now it has become the second-largest metabolic and life-threatening disease worldwide (Chen et al. 2022; Mehmood et al. 2019). Extensive research has shown that hyperuricemia has been linked strongly with kidney disease, especially with hyperuricemic nephropathy and chronic kidney disease (CKD) (Ma et al. 2022; Sato et al. 2019). HUA could increase the risk of kidney diseases and give the risk of death and deterioration of renal function in patients (Kang and Nakagawa 2005). A lot of research demonstrated a positive association between elevated UA levels and prevalent and new-onset CKD (Li et al. 2014). Hence, HUA is considered as a key pathogenic factor for the occurrence, development, and prognosis of most acute and chronic kidney diseases (Toda et al. 2014). However, most urate-lowering drugs including febuxostat, benzbromarone, and allopurinol have their own clinical limitations and adverse reactions in the treatment of HUA nowadays (Strilchuk et al. 2019). Therefore, there is an urgent need to develop safer and more effective agents against HUA.

**Fig. 1:**
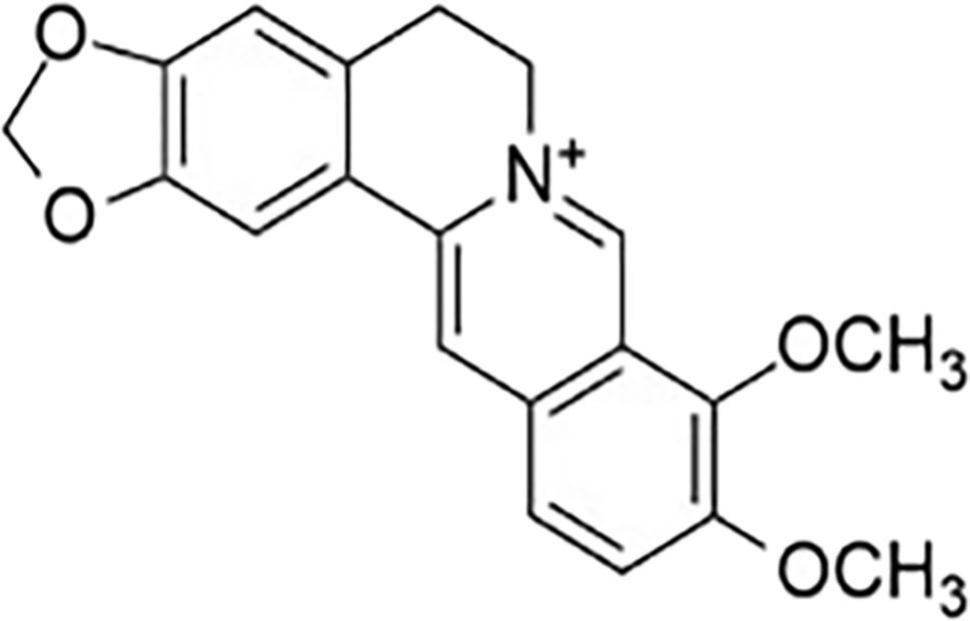
Chemical structure of BBR

The traditional Chinese medicine is known to be characterized by low toxicity and few adverse reactions. In recent years, traditional Chinese medicine has made certain achievements in the treatment of HUA. In other words, many Chinese Medical Materia were reported to be effective for HUA, such as *Fraxini Cortex*, *Phellodendri Cortex*, *Gardenia jasminoides*, and *Poria cocos *(Zhou et al. 2018; Liu et al. 2021; Kong et al. 2004). *Phellodendri Cortex* has long been used to treat HUA and gout (Xu et al. 2021a). Berberine (BBR, the chemical structure is depicted in Fig. 1), an effective drug with anti-inflammatory properties, is an isoquinoline alkaloid as well as a characteristic compound derived from *Phellodendri Cortex *(Zych et al. 2020). And BBR has been reported to have widely pharmacological activities, especially immune regulation, antioxidant, anti-inflammatory, anticancer, and antiviral activities (Ayati et al. 2017). More recently study conducted by Lin et al. documented that berberrubine, one of the major metabolites of BBR, exerts anti-hyperuricemic effect through regulating the function of urate transporter and inhibiting the JAK2/STAT3 signaling pathway in HUA model mice (Li et al. 2021). In our previous study, it was demonstrated that BBR effectively suppressed HUA and improved kidney injury effectively via inhibiting the activation of NLRP3 inflammasome (Li et al. 2021).

Inflammasomes are protein complexes that respond to some pro-inflammatory signals and induce the activation of Caspase-1 that can cleave pro-IL-1β and pro-IL-18, resulting in generating their mature inflammatory counterparts, which play a critical role in the pro-inflammatory and pro-apoptotic effect. Furthermore, inflammasomes can also induce a type of special cell death known as pyroptosis, which is caused by Caspase-1-mediated cleavage of the protein Gasdermin-D (McCarty et al. 2021). It has been reported that NLRP3 was one of the most widely studied inflammasomes (Gross et al. 2011). The activation of NLRP3 seems to contribute significantly to the pathogenesis of HUA and hyperuricemia nephropathy (Wen et al. 2021). Emerging evidence strongly suggests that inhibited NLRP3 is critical in preventing excess inflammatory-damage accumulation and impaired kidney function (Han et al. 2021; Xu et al. 2021b).

Therefore, in order to explore whether NLRP3 is a direct target of BBR in alleviating uric-induced damage and the protective effect of BBR on kidney mediated by cell pyroptosis, we conducted a comprehensive study to explore the effect of BBR on HK-2 cells stimulated by UA in vitro.

## Materials and methods

### Drugs and chemicals

Berberine (BBR, purity ≥ 98%) was supplied by Meilun Biotechnology Co., Ltd. (Dalian, China). Uric acid (UA, purity ≥ 99%) was acquired from Sigma-Aldrich (St. Louis, MO, USA). Z-YVAD-FMK (ZY) was obtained from ApexBio (Houston, USA). Enzyme-linked immunosorbent assay (ELISA) kits for human IL-1β and IL-18 were supplied by Shanghai Enzyme-linked Biotechnology Co., Ltd. (Shanghai, China). The LDH assay kit was obtained from Nanjing Jiancheng Bioengineering Institute (Nanjing, China). Antibodies against Caspase1 (DF6148), IL-1β (AF5103), IL-18 (DF6252), and β-actin (AF7018) were purchased from Affinity Biosciences (Cincinnati, OH, USA). Antibodies against NLRP3 (ab263899), GSDMD (ab210070) were acquired from Abcam (Cambridge, UK). Antibodies against ASC (WL02462), cleaved-Caspase1 (cl-Caspase1, WL03450), cl-Caspase3 (WL02117), cl-Caspase9 (WL02117), BAX (WL01637), and BCL-2 (WL01556) were supplied by Wanshi Biological Technology Co., Ltd. (Shenyang, China). Horseradish peroxidase (HRP)-conjugated secondary antibodies were obtained from EarthOx, LLC (CA, USA). And siRNA was offered by General Biological Systems Co., Ltd. (Anhui, China). Lipofectamine®^2000^ Reagent was obtained from Invitrogen Co., Ltd. (Carlsbad, CA, USA). Opti-MEM^®^ I Reduced Serum Medium was offered by Gibco Co., Ltd.

### Cell culture

The human kidney proximal tubular epithelial cell line (HK-2) was purchased from Green Flag Biotechnology Development Co., Ltd. (Shanghai, China), and grown in DMEM F12 medium containing 10% FBS and 1% penicillin–streptomycin. HK-2 cells were maintained at 37 °C in a 5% CO_2_ atmosphere at 100% humidity.

### Cell couting kit-8 (CCK-8) assay

HK-2 cells were plated at a density of 4.5 × 10^4^ cells/well in 96-well plates and were cultured overnight. Then HK-2 cells were treated with BBR (0, 2.5, 5, 10, 20, 40, 80, 100 μM) or UA (0, 5, 10, 20, 40, 60 mg/dL) for 24 h and 48 h, and then 10 ul CCK8 solution were added to each well. After an hour, the absorbance of the sample at 490 nm was detected by the microplate reader and then cell viability was calculated.

### Lactate dehydrogenase assay

Lactate Dehydrogenase (LDH) activities were measured by LDH assay kit. Briefly, HK-2 cells (2.5 × 10^4^ cells/well) were cultured in a 24-well plate for 24 h, and then cells were treated with the complete medium of UA or UA + BBR for 24 h. Then the cell samples were collected and operated according to the instructions of the manufacturers.

### Assesment of IL-1β and IL-18 by ELISA

The cells were treated as described in Lactate Dehydrogenase Assay previously and the levels of inflammatory cytokines including IL-1β and IL-18 were detected using an ELISA kit according to the manufacturer’s protocol.

### Real-time quantitative PCR (RT-PCR)

The total RNA was extracted from HK-2 cells using TRIzol reagent (Invitrogen, USA) and determined the purity analysis. The cDNA was synthesized with a reverse transcription kit (Vazyme, Nanjing, China). The conditions for PCR amplification were as described (Zheng et al. 2021). The relative mRNA level was normalized to β-actin. The primers synthesized by General Biological Systems (AnHui) company for RT-PCR were listed in Supplementary Material Table S1.

### Western blot analysis

Total cell proteins were lysed in 200 μL RIPA buffer containing. And the protein concentration was measured by the BCA protein assay kit (Bio-Rad, Hercules, CA). Next, the proteins were separated by 10%, 12%, or 15% SDS-PAGE, electroblotted onto PVDF membranes. Then the membrane was blocked with 5% nonfat milk for 90 min and washed 4 times with TBST for 8 min each time. We incubated membranes with appropriate primary antibodies against NLRP3 (1:1000), Caspase1 (1:1000), cl-Caspase1 (1:1000), ASC (1:500), IL-18 (1:1000), IL-1β (1:1000), GSDMD (1:1000), cl-Caspase3 (1:1000), cl-Caspase9 (1:1000), BAX (1:1000), BCL-2 (1:1000), and β-actin (1:1000), overnight at 4 °C. After four 8-min washes, the membranes were incubated with secondary antibody (1:3000) for 60 min. After washing with TBST, the membranes were visualized using ECL reagents (Affinity, OH, USA) and a gel imaging system (Tanon Science & Technology Co., Ltd., China).

### NLRP3 siRNA transfection

NLRP3 small interfering RNA (siRNA) and relative negative control siRNA were purchased from General Biological Systems Co., Ltd (Anhui, China). Using pre-screening by PCR and western blot, the most efficient siRNA of NLRP3 was confirmed. The primer sequences and validation details were provided in Supplementary Materials. Firstly, according to the standard protocol, transfection was performed using Lipofectamine 2000 Reagent at the concentration of 80 nM when the density of HK-2 cells reached 40%. Then the cells were incubated with BBR and UA after 24 h. After the cells were utilized for 24 h, we collected cells for further investigations.

### Statistical analysis

All data were expressed as mean ± SD and SPSS 26.0 software was used for statistical analysis. Differences between multiple treatment groups were evaluated by One-way ANOVA followed by Bonferroni or Dunnet T3 test. Graphical analyses were performed using GraphPad Prism software (ver. 8.02; GraphPad, CA, USA). A *P* value less than 0.05 was considered statistically significant.

## Results

### Cytotoxicities of BBR and UA in HK-2 cells

**Fig. 2:**
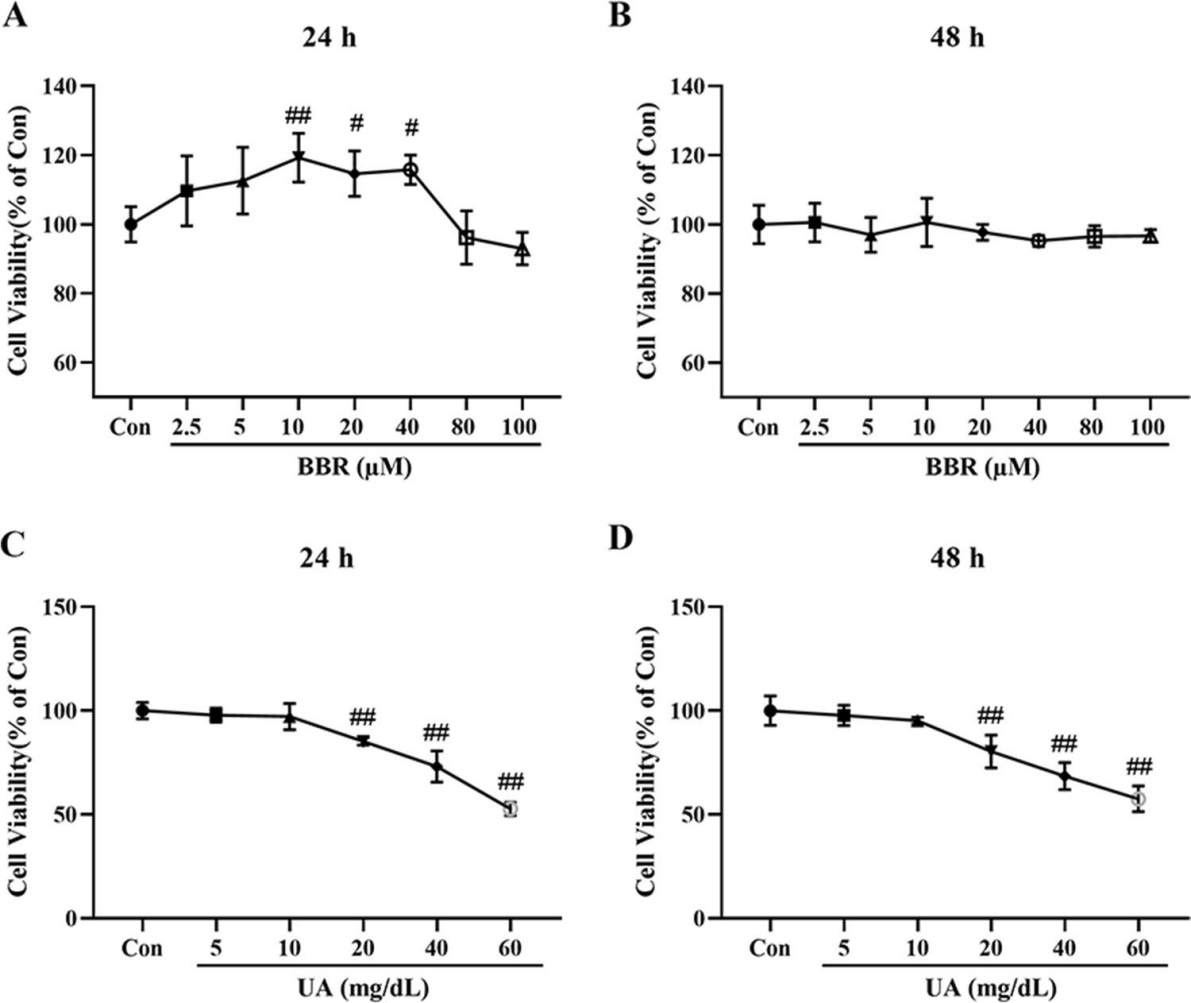
Cytotoxicities of BBR and UA in HK-2 cells. **A** and **B** Effects of treatment with BBR (2.5, 5, 10, 20, 40, 80, 100 μM) for 24 and 48 h on the viability of HK-2 cells. **C** and **D** Effects of treatment with UA (5, 10, 20, 40, 60 mg/dL) for 24 and 48 h on the viability of HK-2 cells. Values represent the means ± SD (*n* = 6). BBR, berberine; UA, uric acid. ^#^*P* < 0.05, ^##^*P* < 0.01 vs Con

To evaluate the effect of BBR on HK-2 cells in vitro, the CCK-8 assay was used to detect the cell viability. As exhibited in Fig. 2A, 10, 20, and 40 μM BBR dramatically increased cell viability following stimulation by BBR 24 h when compared with the Con group. In addition, other different concentrations of BBR did not show significant cytotoxicities to HK-2 cells when incubated for 24 h. Similarly, BBR at the concentrations 2.5 μM up to 100 μM showed no adverse effects on HK-2 cell viability when incubated for 48 h (Fig. 2B). Moreover, to study the effects of UA on HK-2 cells, the cells were cultured in different concentrations of UA (5, 10, 20, 40, 60 mg/dL) for 24 h and 48 h. As shown in Fig. 2C and D, UA induced cell death in a dose-dependent manner when they were treated for 24 h and 48 h. Compared to the Con group, a marked decrease was seen in cell viability after treatment with UA (20, 40, 60 mg/dL).

### Effects of BBR on the level of inflammatory factors and LDH in HK-2 cells

**Fig. 3:**
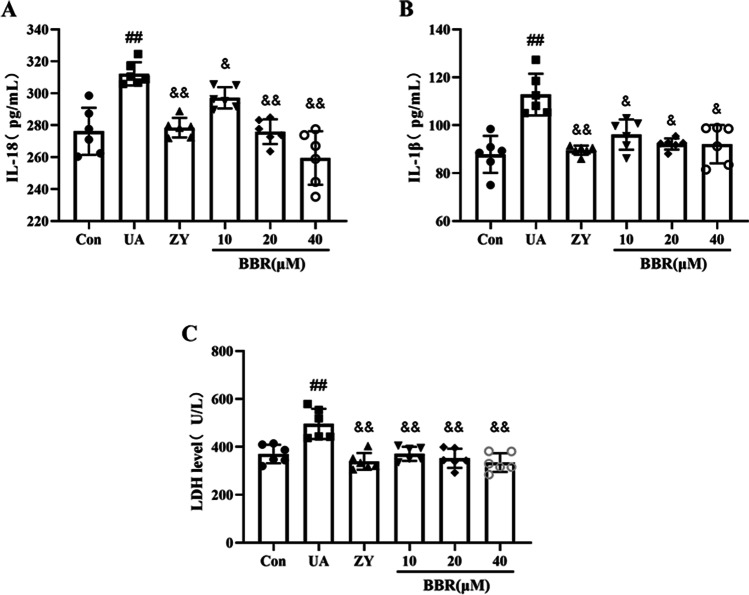
Effects of BBR on the level of inflammatory factors and LDH in HK-2 cells. **A** and **B** Effects of BBR on the level of inflammatory factors in HK-2 cells. **C** Effects of BBR on the level of LDH in HK-2 cells. Values represent the means ± SD (*n* = 6). BBR, berberine; UA, uric acid (20 mg/dL); ZY, Z-YVAD-FMK, (10 μM). ^##^*P* < 0.01 vs Con; ^&^*P* < 0.05, ^&&^*P* < 0.01 vs UA

To explore the protective effects of BBR on UA-induced HK-2 cells, the levels of inflammatory factors IL-1β and IL-18 were measured by ELISA. As shown in Fig. 3A and B, the levels of inflammatory factors IL-1β and IL-18 were enhanced markedly in the UA group when compared with the Con group. Compared to the UA group, a marked decrease was seen in the expression levels of IL-1β and IL-18 after treatment with ZY and BBR (10, 20, 40 μM). It is generally assumed that the destabilization of the plasma membrane is a key feature of pyroptotic cell death and LDH is a reference index of cell membrane integrity and also serves as a marker of lysate cell death (Gurunathan et al. 2014). Hence, we determined the levels of LDH in order to assess whether UA induced cell injury. Concentrations of LDH were higher in the UA group than in the Con group (Fig. 3C). Meanwhile, we found that after ZY and BBR treatment, a significant decrease in LDH levels was observed. Together these results suggest that treatment with BBR suppressed the expressions of inflammatory factors and LDH levels in HK-2 cells, which is helpful to attenuate cell damage induced by UA.

### Effects of BBR on apoptosis-related genes in HK-2 cells

**Fig. 4:**
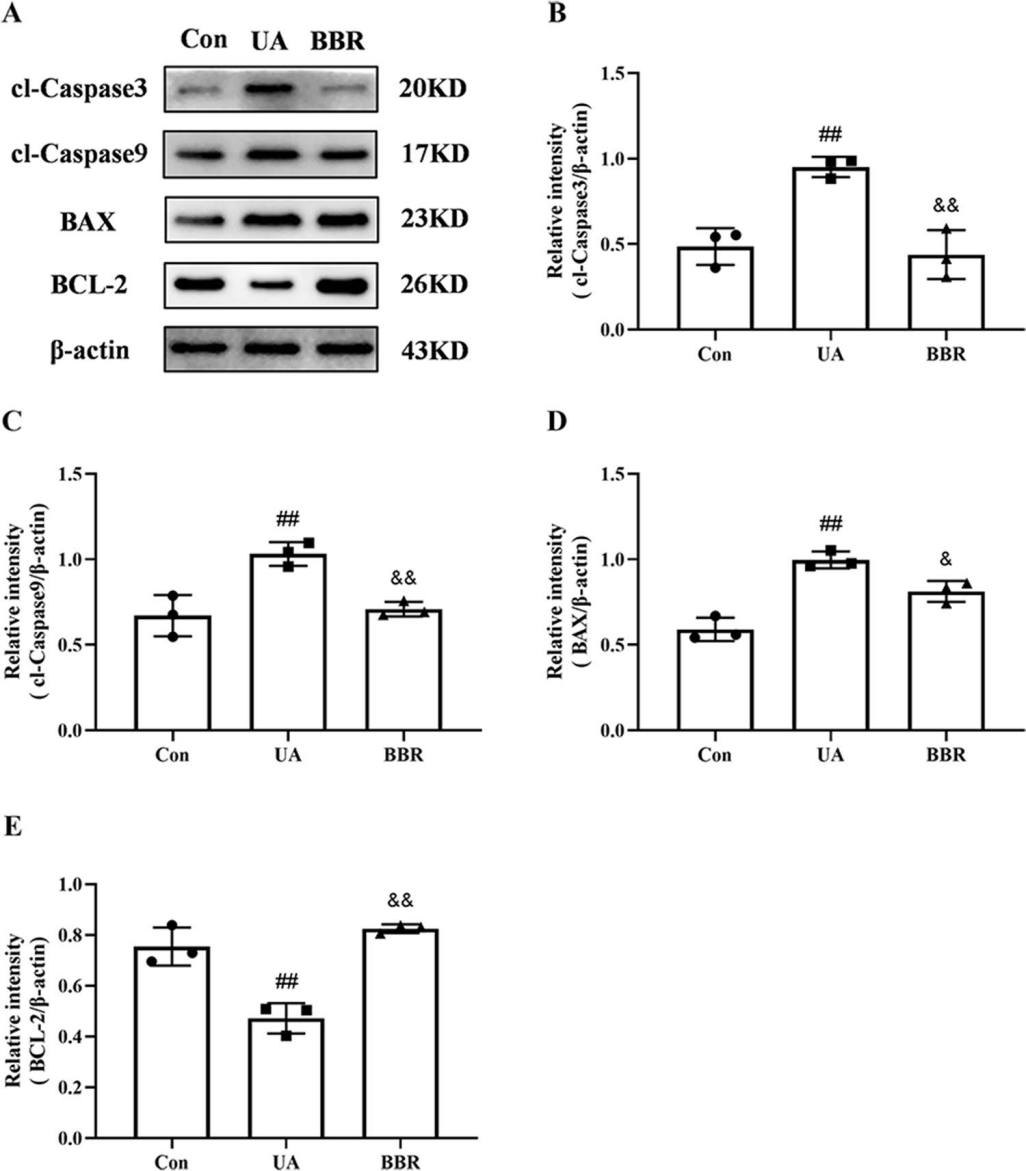
Effects of BBR on apoptosis-related genes in HK-2 cells. **A** Western blot analyses of cl-Caspase3, cl-Caspase9, BAX, BCL-2, and β-actin. **B** Intensity of cl-Caspase3, cl-Caspase9, BAX, and BCL-2 relative to β-actin. Values represent the means ± SD (*n* = 3). BBR, berberine (20 μM); UA, uric acid (20 mg/dL); cl-Caspase3, cleaved-Caspase3; cl-Caspase9, cleaved-Caspase9. ^##^*P* < 0.01 vs Con; ^&^*P* < 0.05, ^&&^*P* < 0.01 vs UA

The western blot results of the expression of cl-Caspase3, cl-Caspase9, BAX, and BCL-2 were shown in Fig. 4. Compared with the Con group, UA could significantly increase the expression of cl-Caspase3, cl-Caspase9, and BAX (Fig. 4B–D). Conversely, the expression level of BCL-2 was dramatically reduced in the above comparison (Fig. 4E). And these abnormal changes were reversed by BBR treatment. As anticipated, there were significant degrees of protection against UA-induced cell apoptosis in the BBR group compared with the UA group.

### Effect of BBR on NLRP3 pathway in HK-2 cells

**Fig. 5:**
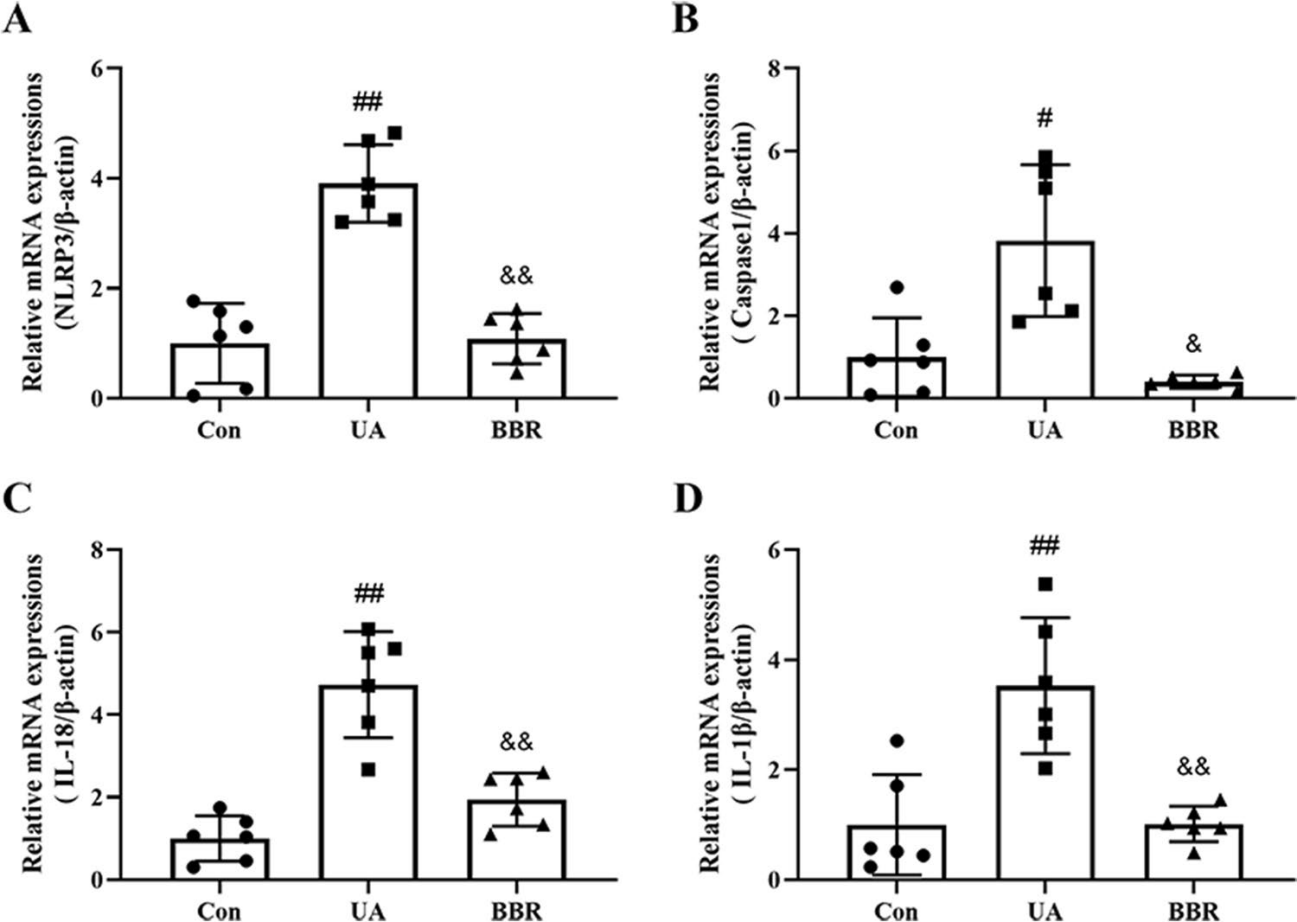
Effect of BBR on NLRP3 pathway in HK-2 cells using RT-PCR. **A**–**D** Relative mRNA expression levels of NLRP3, Caspase1, IL-18, and IL-1β. Values represent the means ± SD (*n* = 6). BBR, berberine (20 μM); UA, uric acid (20 mg/dL). ^#^*P* < 0.05, ^##^*P* < 0.01 vs Con; ^&^*P* < 0.05, ^&&^*P* < 0.01 vs UA

**Fig. 6:**
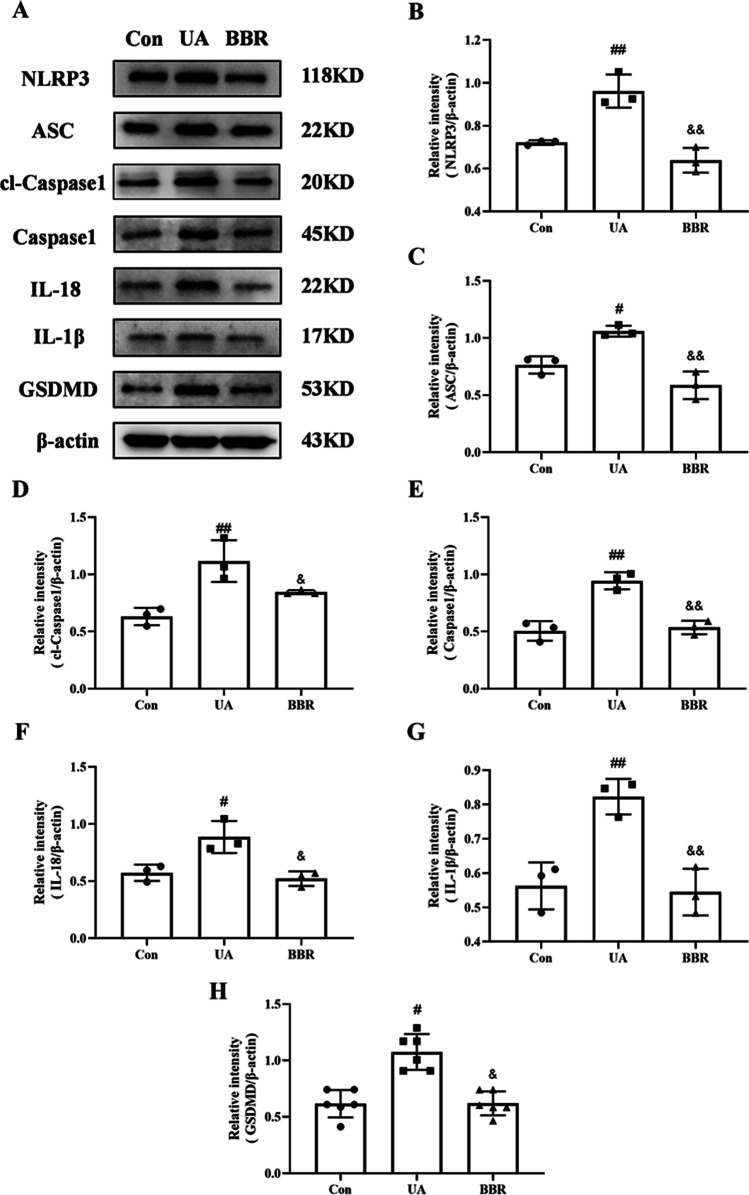
Effect of BBR on NLRP3 pathway in HK-2 cells using Western blot. **A** Western blot analyses of NLRP3, ASC, cl-Caspase1, Caspase1, IL-18, IL-1β, GSDMD, and β-actin. **B**–**H** Intensity of NLRP3, ASC, cl-Caspase1, Caspase1, IL-18, IL-1β, and GSDMD relative to β-actin. Values represent the means ± SD (*n* = 3). BBR, berberine (20 μM); UA, uric acid (20 mg/dL); cl-Caspase1, cleaved-Caspase1. ^#^*P* < 0.05, ^##^*P* < 0.01 vs Con; ^&^*P* < 0.05, ^&&^*P* < 0.01 vs UA

To explore the regulatory mechanism of BBR in HK-2 cells induced by UA, the NLRP3 pathway-related markers were determined by RT-PCR and western blot. The RT-PCR results exhibited that the mRNA levels of NLRP3, Caspase1, IL-18, and IL-1β were markedly upregulated as compared to that of the Con group, while this upregulation was dramatically decreased by BBR treatment (Fig. 5). Additionally, the western blot result revealed that the expression of NLRP3, ASC, cl-Caspase1, Caspase1, IL-18, IL-1β, and GSDMD increased significantly in the UA group compared with the Con group and BBR treatment was capable of depressing the protein expression of the above indicators (Fig. 6). Hence, these data confirmed that BBR alleviated the UA induced cell damage by modulating the NLRP3 pathway.

### Effects of BBR on the level of inflammatory factors and LDH in HK-2 cells following NLRP3 siRNA knockdown

**Fig. 7:**
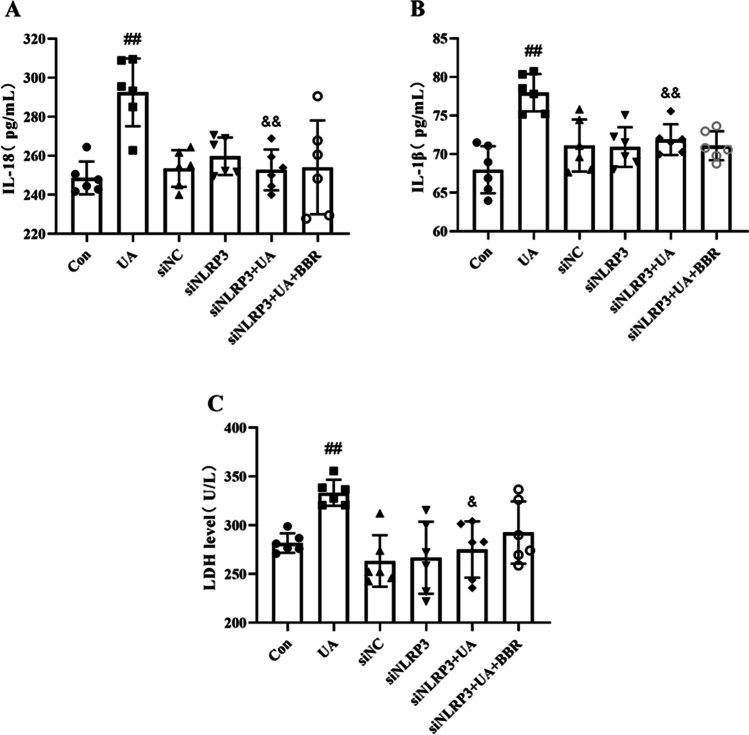
Effects of BBR on the level of inflammatory factors and LDH in HK-2 cells following NLRP3 siRNA knockdown. **A** and **B** Effects of BBR on the level of inflammatory factors following NLRP3 siRNA knockdown. **C** Effects of BBR on the level of LDH following NLRP3 siRNA knockdown. Values represent means ± SD (*n* = 6). BBR, berberine (20 μM); UA, uric acid (20 mg/dL). ^##^*P* < 0.01 vs Con; ^&^*P* < 0.05, ^&&^*P* < 0.01 vs UA

NLRP3 plays a critical role in HUA and activation of NLRP3 leads to ASC specks accumulated and Caspase-1 recruited to activated, which further promotes maturation of IL-1β and IL18. To confirm whether the protective effect of BBR was dependent on NLRP3, siRNA was applied to down-regulate NLRP3 expression in HK-2 cells. As demonstrated in Fig. 7, the contents of inflammatory factors (IL-18, IL-1β) and the LDH level in HK-2 cells induced by siNLRP3 + UA were significantly decreased as compared to the UA group. Meanwhile, we found that no effect on the contents of inflammatory factors and LDH level in HK-2 cells occurred siNLRP3 + UA + BBR group with the siNLRP3 + UA group.

### Effect of BBR on NLRP3 pathway in HK-2 cells following NLRP3 siRNA knockdown

**Fig. 8:**
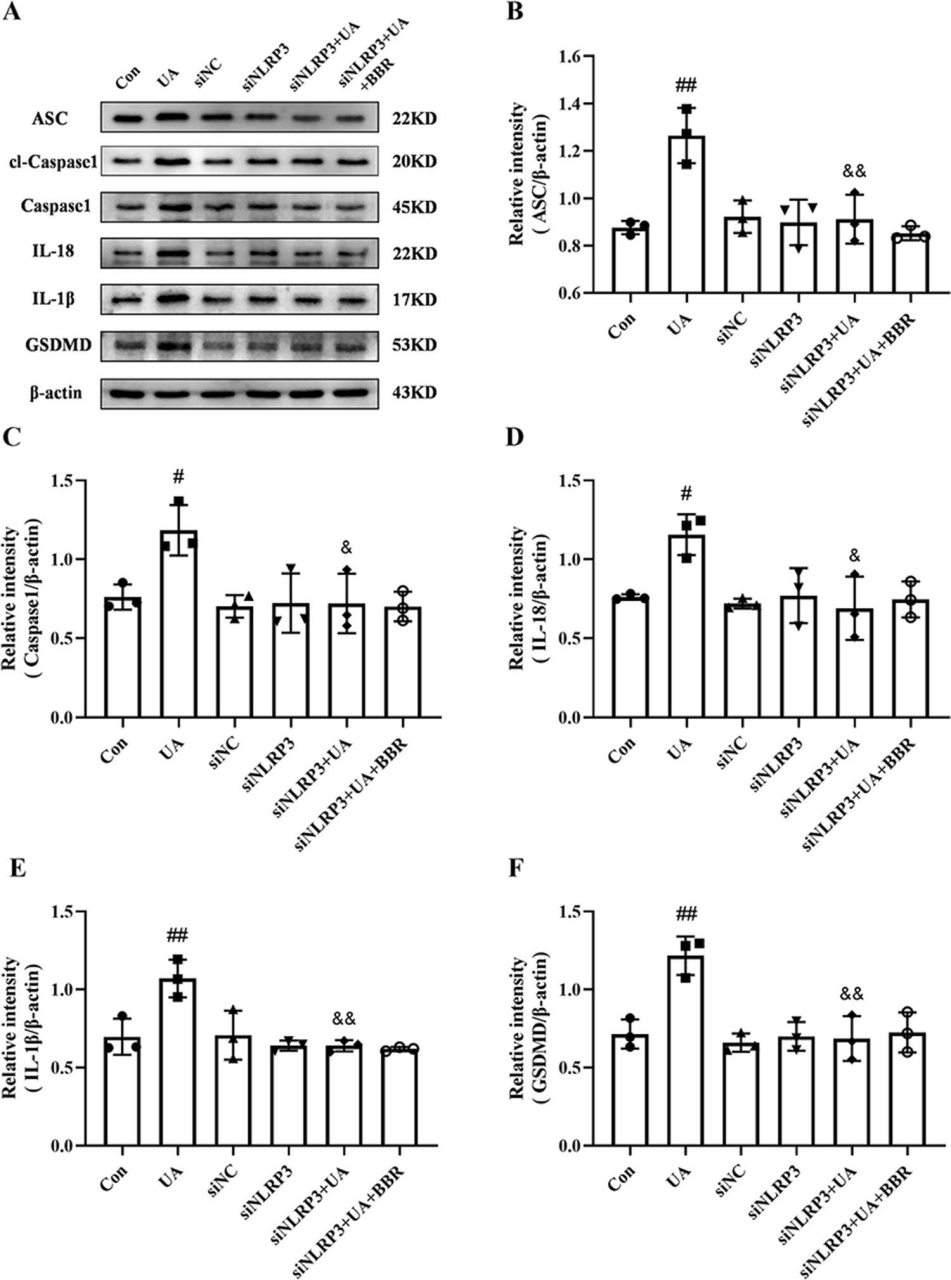
Effect of BBR on NLRP3 pathway in HK-2 cells following NLRP3 siRNA knockdown. **A** Western blot analyses of ASC, cl-Caspase1, Caspase1, IL-18, IL-1β, GSDMD, and β-actin. **B**–**G** Intensity of ASC, cl-Caspase1, Caspase1, IL-18, IL-1β, and GSDMD relative to β-actin. Values represent the means ± SD (*n* = 3). BBR, berberine (20 μM); UA, uric acid (20 mg/dL); cl-Caspase1, cleaved-Caspase1. ^#^*P* < 0.05, ^##^*P* < 0.01 vs Con; ^&^*P* < 0.05, ^&&^*P* < 0.01 vs UA

To further analyze whether the protective effect of BBR against injury was mediated by the NLRP3 pathway, the expression of NLRP3 downstream proteins was determined after transfection with siNLRP3. After NLRP3 silence, a dramatically decrease in the expression of ASC, Caspase1, cl-Caspase1, IL-18, IL-1β, and GSDMD was observed between UA and siNLRP3 + UA group. Furthermore, compared with the siNLRP3 + UA group, no significant changes in the expression of ASC, Caspase1, cl-Caspase1, IL-18, IL-1β, and GSDMD after BBR treatment. The result showed that the regulations of BBR on UA production were blocked through NLRP3 siRNA (Fig. 8).

## Discussion

UA, the hallmark of HUA, serves as an antioxidant to neutralize various oxidants such as H_2_O_2_ and NO of the body and reduce their toxic effects (Lane et al. 2014). On the other hand, UA serves as a prooxidant when excessive UA concentration in the blood, which could induce oxidative stress, mitochondrial dysfunction, promote the release of inflammatory factors and the activation of renin-angiotensin, resulting in damaged vascular endothelial cells, the vascular proliferation of smooth muscle cells, and renal interstitial fibrosis (Liu et al. 2021). Choosing the suitable concentrations and incubation time of UA to establish models are of great value for our study. According to previous experiments, 20 mg/dL UA could significantly promote the expression of IL-18 in HK-2 cells after incubating 24 h (see Supplementary Materials for experimental details). Additionally, our experimental results indicated that BBR show no toxic effect on HK-2 cells. But for UA, the cell viability was evidently decreased with raising UA concentration in a concentration-dependent manner. When the concentration of UA was greater than or equal to 20 mg/dL, a significant cytotoxic effect on HK-2 cells was observed. Therefore, 20 mg/dL UA-induced HK-2 cells model was adopted as an HUA model in vitro in this paper.

IL-18 and IL-1β, the predominant members of the interleukin-1 family, are important cytokines regulating inflammation and immune response, which can be used as markers of the degree of inflammation (Dinarello 2013). HUA often leads to aggravating renal damage, frequently accompanied by inflammatory responses. Generally speaking, high concentrations of LDH enzyme mainly exist in the liver, kidney, heart, and other tissues, and once they were damaged, the LDH levels significantly increased in the serum (Heidari Beigvand et al. 2021). Elevated LDH has also been shown to increase the risk for acute kidney injury (Personett et al. 2019). In this study, our results showed that the IL-18, IL-1β, and LDH levels of the UA group was a statistically significant increase when compared to the Con group. And a dramatic reduction of the IL-18, IL-1β, and LDH levels was seen with BBR or ZY treatment. Notably, the study revealed that a BBR dosage of 40 μM gave the best results compared to other dosages. Indeed, the effect of BBR displays little difference between 20 and 40 μM. Thus, 20 μM BBR was used in subsequent RT-PCR and Western blot experiments. In short, our results revealed that BBR did in fact inhibit inflammatory cytokines and LDH release to alleviate cell damage induced by UA.

Although the pathophysiological mechanism of HUA remains to be fully elucidated, apoptosis and inflammation are partly known for their involvement in the pathogenesis of HUA. There is a strong association between UA and cell apoptosis that has previously been reported (Deng et al. 2021). According to Zhao et al., Withaferin A protects against UA-induced apoptosis in NRK-52E cells that it markedly upregulated the expression of pro-apoptosis genes including BAX, cl-caspase3, and cl-caspase9, accompanied by down-regulated the expression of anti-apoptosis BCL-2 (Zhao et al. 2021). Consistent with Zhao’s study, BBR also effectively prevents UA-induced cell apoptosis by promoting the expression of anti-apoptotic proteins such as BCL-2 and inhibiting the expression of antiapoptotic protein BAX, cleaved caspase3, and cleaved caspase9. Our findings suggested that BBR blocks UA-induced apoptosis in HK-2 cells to relieve the cell injury.

As an independent risk factor for the occurrence and progression of clinical chronic kidney disease, the increase of uric acid level can cause kidney damage through the activation of oxidative stress and inflammatory response (Li et al. 2020). When it comes to inflammation, it is inevitable for us to talk about the classic inflammatory signaling pathway-NF-KB signaling pathway. Another inflammation core pathway-NLRP3 signaling pathway is often neglected because it was less understood in the past*.* However, the growing body of research has helped to improve our understanding of this signaling pathway, which has attracted more and more attention in the last decades. The researchers reported that UA aggravates ROS generation to promote the NLRP3 activation and pyroptosis in the myocardial ischemia–reperfusion (MI/R) model, while inflammasome inhibitors and ROS scavengers ameliorate MI/R damage (Shen et al. 2021). Once NLRP3 is activated, inflammasome assembly occurs, triggering the release of significant quantities of proinflammatory cytokines. In other words, NLRP3 oligomerizes and binds to ASC that leads to the activation of Caspase1, which subsequently cleaves pro-IL-1β and pro-IL-18 to promote maturation and release of inflammatory cytokines IL-1β and IL-18, and inflammatory cell death, pyroptosis (Sharma and Kanneganti 2021). To our knowledge, the key downstream proteins of the NLRP3 signaling pathway also include the GSDMD protein, which is appreciated as a symbol as well as the key executioner of pyroptosis (Gao et al. 2018). Of note, unlike apoptosis, autophagy, and necrosis, pyroptosis is a unique type of cell death that is accompanied by a strong inflammatory response and closely coupled with increased membrane porosity, cellular expansion, and DNA damage (Qiu et al. 2018). Hoffman et al. found that urate crystals induced a reduced inflammatory response, reducing caspase-1 activation and IL-1β release in NLRP3 knockout mice (Hoffman et al. 2010). In addition, a growing body of evidence suggested that dihydroberberine, the derivative of BBR, could inhibit NLRP3 inflammasome activation to mitigate kidney inflammation and dysfunction (Xu et al. 2021a; Lin et al. 2021). In the present study, the result showed that UA significantly up-regulate the expression levels of NLRP3, Caspase1, IL-18 and IL-1β and result in the over-expression of NLRP3, ASC, Caspase1, cl-Caspase1, IL-18, IL-1β and GSDMD. Conversely, the data from our study showed that BBR could suppress the aberrant mRNA level of NLRP3, Caspase1, IL-18, and IL-1β. Besides, we found that BBR effectively inhibits the aberrant protein expression of NLRP3, ASC, Caspase1, cl-Caspase1, IL-18, IL-1β, and GSDMD. Hence, these data confirmed that BBR exerts its protective effect via suppressing the NLRP3 pathway.

Nevertheless, the NLRP3 inflammasome-related signaling pathways are quite complex, and further experiments are required to reveal the specific mechanism on how BBR regulates NLRP3 signaling pathway to affect UA-induced cell injury in HK-2 cells. Thus, in order to confirm whether the protective effect of BBR was dependent on NLRP3, siRNA was applied to down-regulate NLRP3 expression in HK-2 cells. Subsequently, we observed the levels of inflammatory factors (IL-8, IL-1β), LDH levels and NLRP3 pathway-related proteins (ASC, Caspase1, cl-Caspase1, IL-18, IL-1β, and GSDMD) following siNLRP3 transfection. Interestingly, no significant differences were found in all indices above. As a consequence, these results suggested that NLRP3 deficiency did not affect the effect of BBR. Furthermore, these data demonstrated that BBR exerts its antioxidant action by mediating the NLRP3 pathway.

Based on the current achievements, we further identified that UA-induced apoptosis and pyroptosis of HK-2 cells and BBR can effectively alleviate cell injury by inhibiting the NLRP3 signaling pathway.

## Conclusion

In summary, our results proved that BBR has strong therapeutic potential for UA-induced renal inflammation, which also served as a new complementary treatment for UA-related complications, such as HUA and hyperuricemia nephropathy.

## Supplementary Information

Below is the link to the electronic supplementary material.

Supplementary file1 (DOCX 201 kb)

Supplementary file2 (DOCX 78288 kb)

## Data Availability

The raw data supporting the conclusions of this article will be made available by the authors, without undue reservation.
