# Supplementary material for: Berberine attenuates uric acid-induced cell injury by inhibiting NLRP3 signaling pathway in HK-2 cells
Source: Naunyn Schmiedebergs Arch Pharmacol. 2023 May 17;396(10):2405–16. doi: 10.1007/s00210-023-02451-3 (PMC10497693; doi:10.1007/s00210-023-02451-3)
Supplement: Supplementary file 1 — Supplementary file1 (DOCX 201 kb) [file 210_2023_2451_MOESM1_ESM.docx]

Supplementary Materials

# Supplementary Table

**Table S1. Primers sequences used for quantitative PCR.**

| **Gene name** |  | **Primer (5′-3′)** |
| --- | --- | --- |
| **NLRP3** | Forward | AAGGAAGTGGACTGCGAGAA |
|  | Reverse | AACGTTCGTCCTTCCTTCCT |
| **Caspase1** | Forward | GGCATGACAATGCTGCTACA |
|  | Reverse | TCTGGGACTTGCTCAGAGTG |
| **IL-18** | Forward | TCACCAGAGGTCAGGTGTTC |
|  | Reverse | TCCGGAGTGCAAGTGATTCT |
| **IL-1β** | Forward | AGCTGAGGAAGATGCTGGTT |
|  | Reverse | GTGATCGTACAGGTGCATCG |
| **β-actin** | Forward | TGAAGATCAAGATCATTGCTCCTCCT |
|  | Reverse | GACTCGTCATACTCCTGCTTGCTG |

# Supplementary Data

2.1 Methods

The HK-2 cell line was obtained from American Type Culture Collection (Manassas, USA), and grown in DMEM F12 medium containing 10% FBS and 1% penicillin-streptomycin. HK-2 cells were incubated at 37°C with 5% CO2 in a humidified atmosphere.

2.2 Study on the optimal concentration of UA-induced renal cell injury

Briefly, HK-2 cells (2.5×10^4^ cells/well) were cultured in 24-well plate for 24 h and then cells treated with different concentrations of UA (0, 5, 10, 20, 40, 60 mg/dL) for 24 h or 48 h. Then the cell samples were collected and the levels of IL-18 were detected using ELISA kit according to the manufacturer’s protocol.

2.3 siRNA preparation and transfection

The negative control siRNA and si-NLRP3 gene-specific sequences were synthesized by General Biological Systems (AnHui) company. Lipofectamine®2000 Reagent (Invitrogen, Carlsbad, CA, USA) was used as the transfection agent. Transfection of siRNA was carried out according to the manufacturer’s protocol.

Briefly, when the HK-2 cells confluence about to 40% in six well plates started to transfect. First, 5 μL of Lipofectamine®^2000^ Reagent was mixed with 250 μL Opti-Mem and incubated 5 minutes at room temperature; 8 μLof siRNA was mixed with 250 μL Opti-Mem incubated 5 minutes at room temperature. Then two mixtures were mixed together and the mixture was incubated at room temperature for 20 minutes. Finally, 1.5 mL Opti-Mem added into the mixture and started to transfect. About 6 hours later, changed to normal DMEM F12 medium without penicillin-streptomycin, and the final siRNA concentration was 80 nM.

2.4 Real-time quantitative PCR

Total RNA was isolated from HK-2 cells using the TRIzol reagents (Invitrogen). Immediately, 2 ug RNA using the HiScript II Reverse Transcriptase Kit to synthetic cDNA. Then applying the SYBR fluorescence probe to PCR-amplified the cDNA, the programs were run at stage 1: 95 ℃ for 30 secs; stage 2: followed by 40 cycles at 95 ℃ for 10 secs and 60 ℃ for 30 secs; stage 3: 95 ℃ for 15 secs, 60℃ for 60 secs and 95 ℃ for 15 secs. GAPDH was used as an internal control. Fold change= 2-ΔΔCt, ΔΔCt = (Ct Sample– Ct β-actin) – (Ct Control– Ct β-actin). The primers sequences of genes including β-actin, si-NLRP3 were synthesized by Invitrogen (Carlsbad, CA, UAS). The siRNA sequences were listed in Table S2.

2.5 Western blot analysis

The cells were washed in pre-cold PBS three times, the total protein was extracted and then using the BCA protein assay kit (Bio-Rad, Hercules, CA) to measure the protein concentrations and unify the loading quantities. Subsequently, proteins were separated by 10% SDS polyacrylamide gel electrophoresis (PAGE) and electrophoresis to transfer onto PVDF membranes and washed with TBST twice times. Then blocked for 1 h with PBS containing 5% dried milk powder and incubated overnight at 4°C with primary antibodies. The membranes were then washed in TBST three times, and the appropriate HRP-conjugated secondary antibodies diluted (1:3000) in 5% dried milk incubated 1h, washed with TBST three times and developed.

**Table S2. Primers sequences used for quantitative PCR.**

| **Gene name** |  | **Primer (5′-3′)** |
| --- | --- | --- |
| **siNLRP3-1** | **Forward** | **GAAAUGGAUUGAAGUGAAATT** |
|  | **Reverse** | **UUUCACUUCAAUCCAUUUCTT** |
| **siNLRP3-2** | **Forward** | **AGGAAGAGGAGGAGGAAAATT** |
|  | **Reverse** | **UUUUCCUCCUCCUCUUCCUTT** |
| **siNLRP3-3** | **Forward** | **CGUAAGAAGUACAGAAAGUTT** |
|  | **Reverse** | **ACUUUCUGUACUUCUUACGTT** |
| **β-actin** | **Forward** | **TGAAGATCAAGATCATTGCTCCTCCT** |
|  | **Reverse** | **GACTCGTCATACTCCTGCTTGCTG** |

3. Results

3.1. BBR suppressed the expression of IL-18 in UA-stimulated HK-2 cells

In order to determine the optimal inducing concentration and time, various UA concentrations from 5 mg/dL to 60 mg/dL were employed, separately. IL-18 expression was measured as index of cell injury. As shown in Fig. S1A, compared with Con group, the expression of IL-18 were significantly increased at concentrations equal to or greater than 10 mg/dL UA when incubated for 24 h. And significant increase of IL-18 content was also observed at concentrations equal to or greater than 20 mg/dL UA when incubated for 48 h. Therefore, according to the above experimental research results, the optimal UA concentration and incubate time were initially determined. The optimal concentration and treatment time with UA was 20 mg/dL and 24 h, respectively.


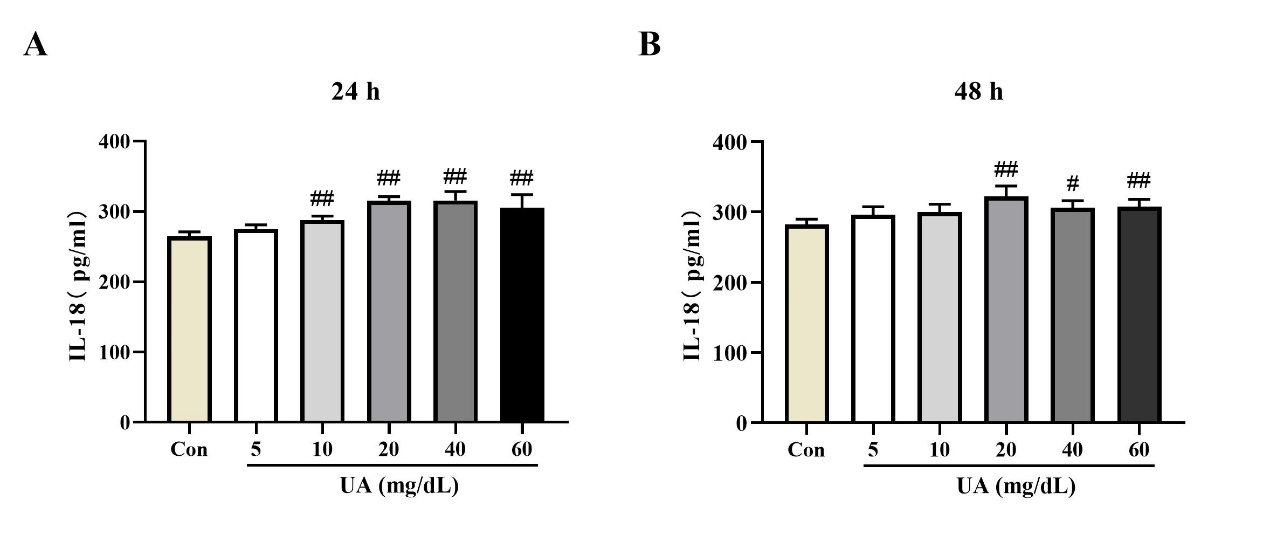


**Fig. S1.** The expression of IL-18 induced by UA in HK-2 cells. (A) The expression of IL-18 induced by UA in HK-2 cells for 24 h. (B) The expression of IL-18 induced by UA in HK-2 cells for 48 h. Values represent the means ± SD (n=6). ^#^ *p* < 0.05, ^##^ *p* < 0.01 vs Con.

3.2 NLRP3 expression were suppressed with NLRP3 siRNA knockdown

The western blot results suggested that after 48 h transfected with three pairs of siRNAs, the protein level of NLRP3 was decreased compared with the siNC group, but only siNLRP3-3 group had significant differences (*p* < 0.05) (Fig. S2A-B). Moreover, the mRNA expression level of NLRP3 in HK-2 cells were down-regulated by transfected siRNAs for 48 h, but only siNLRP3-2 group and siNLRP3-3 group had significant differences (*p* < 0.01) (Fig. S2C). According to the results, the most effective siRNA was siNLRP3-3 which was used in the subsequent studies.


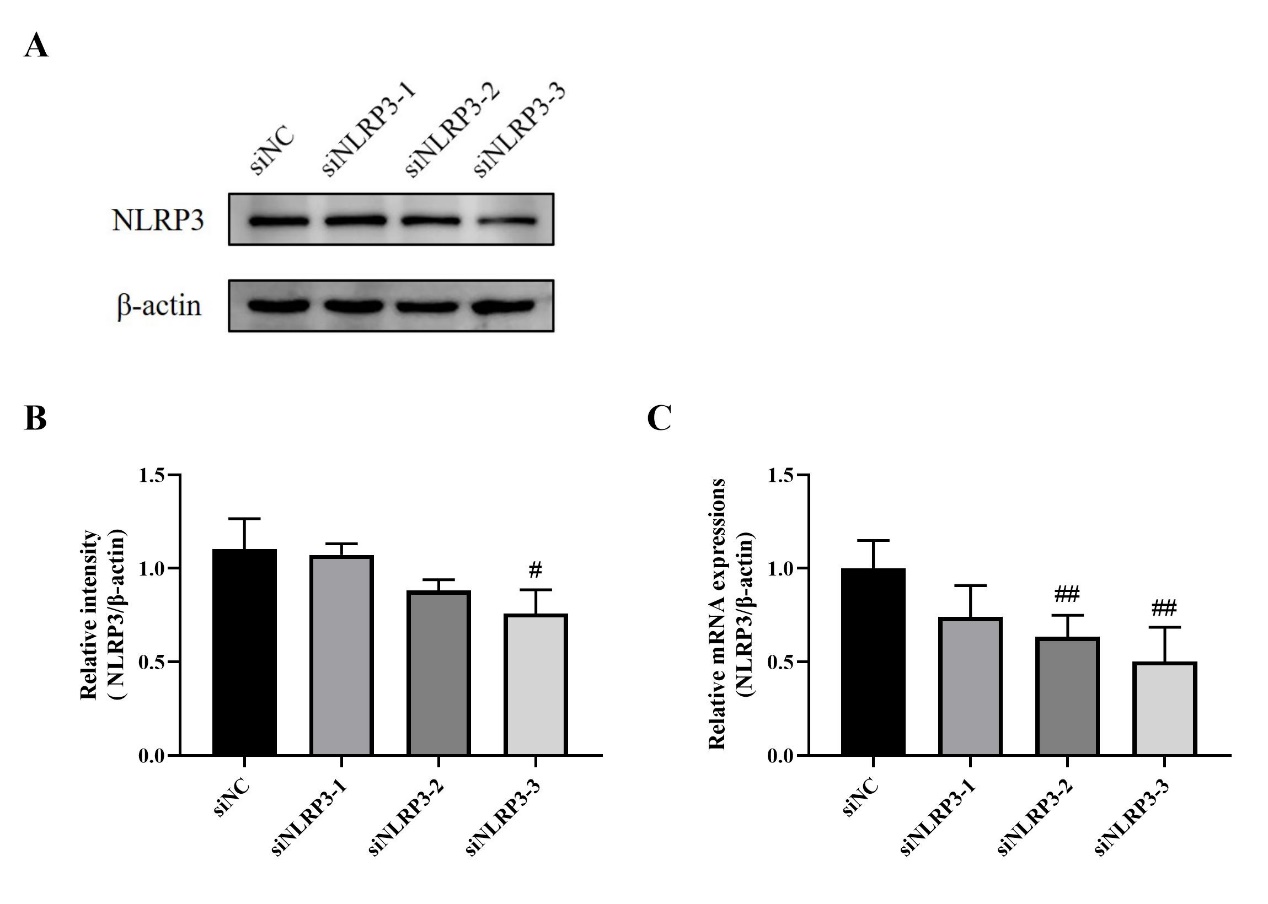


**Fig. S2.** The expression of NLRP3 was suppressed with NLRP3 siRNA. (A) Western blot analysis of relative protein level of NLRP3 for 48 h after transfection with three pairs of siRNAs. (B) Relative intensity of NLRP3 to β-actin. (C) RT-PCR analysis of relative mRNA level of NLRP3 for 48 h after transfection. Values represent the means ± SD (n=3-6). ^#^ *p* < 0.05, ^##^ *p* < 0.01 vs siNC.
