# Supplementary material for: Berberine attenuates uric acid-induced cell injury by inhibiting NLRP3 signaling pathway in HK-2 cells
Source: Naunyn Schmiedebergs Arch Pharmacol. 2023 May 17;396(10):2405–16. doi: 10.1007/s00210-023-02451-3 (PMC10497693; doi:10.1007/s00210-023-02451-3)
Supplement: Supplementary file 2 — Supplementary file2 (DOCX 78288 kb) [file 210_2023_2451_MOESM2_ESM.docx]

**(Fig.4A) β-actin 43kDa**


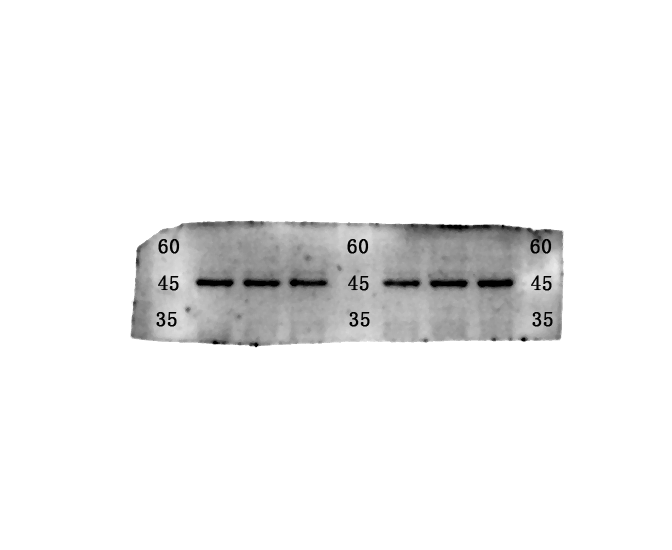


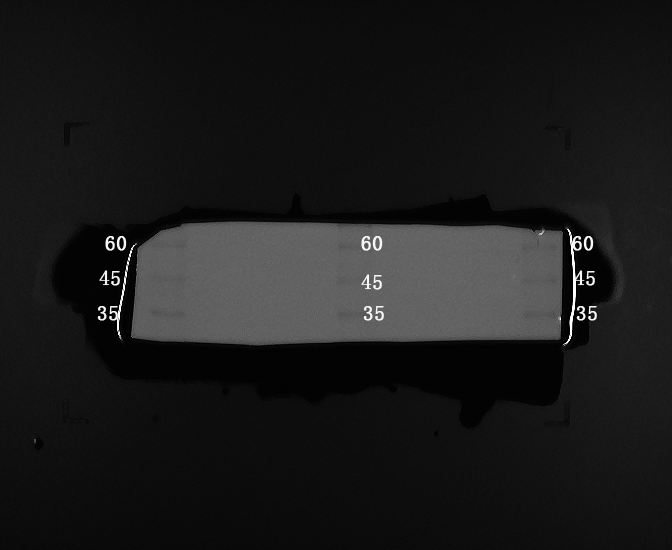


**Repeat 1 (left)**


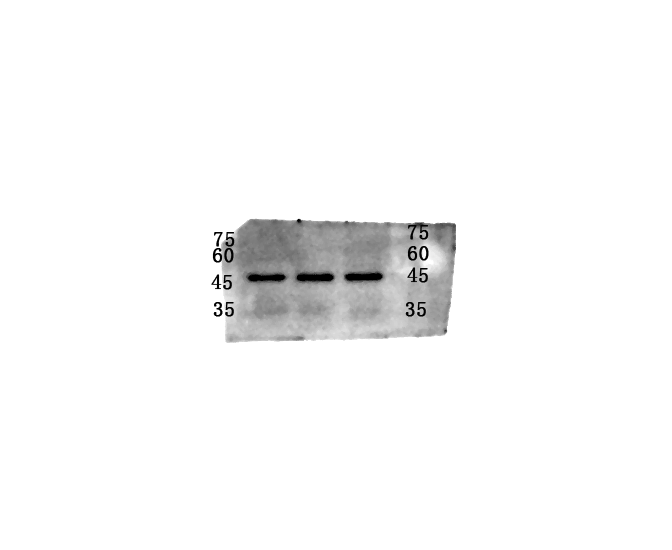


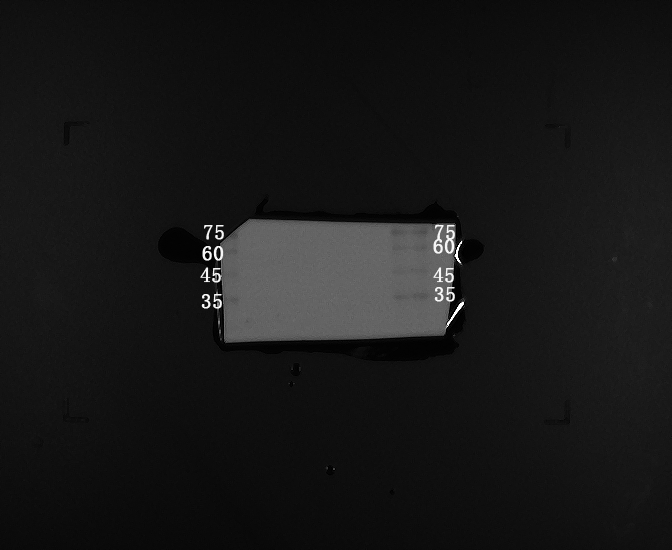


**Repeat 2**


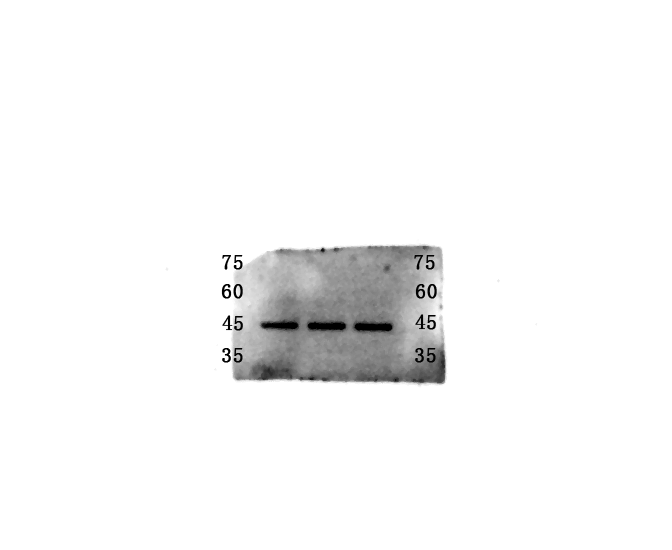


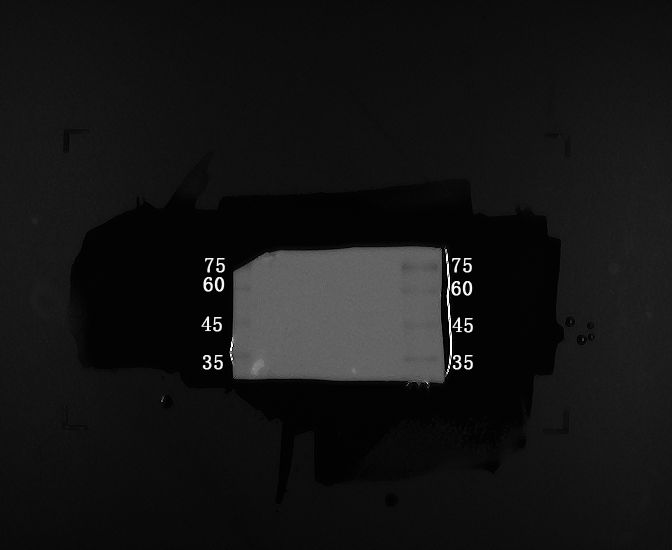


**Repeat 3**

**cl-Caspase3 20kDa**


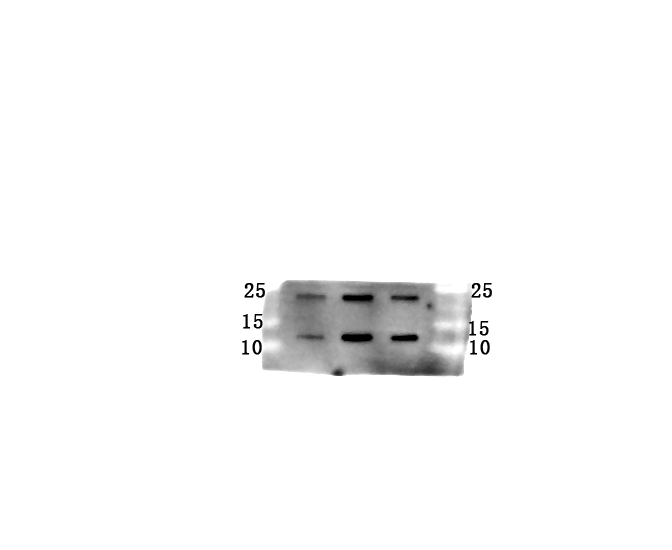


**Repeat 1**


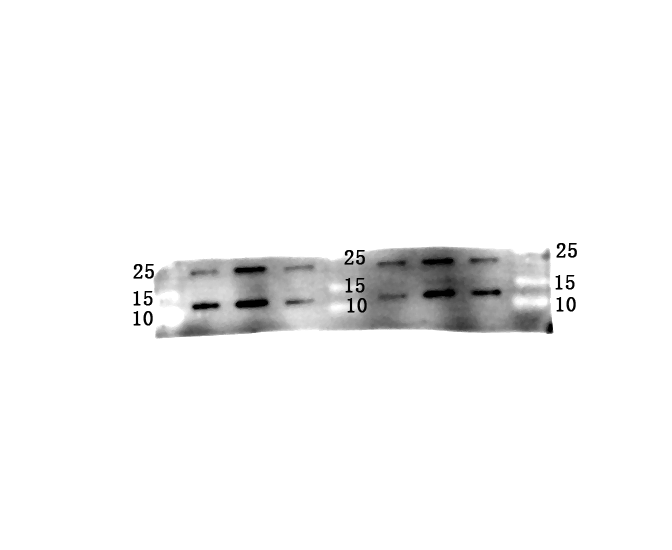


**Repeat 2+Repeat 3**

**cl-Caspase9 17kDa**


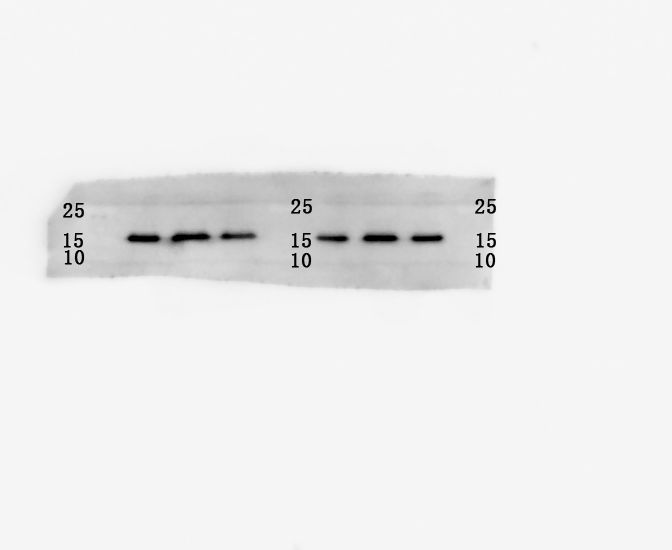


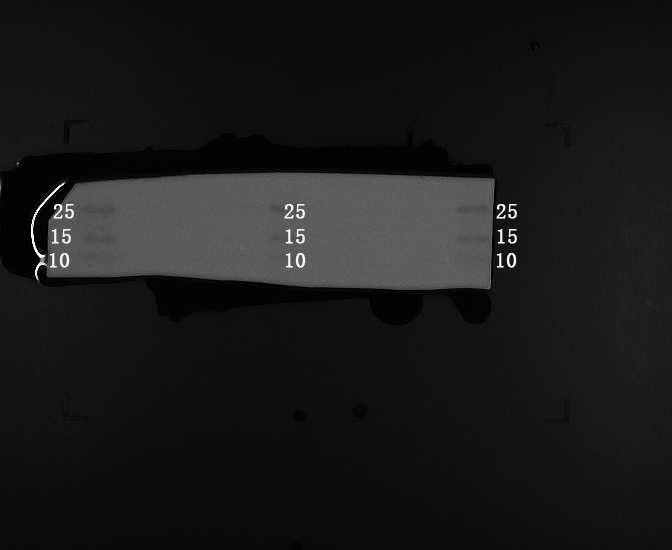


**Repeat 1+Repeat 2**


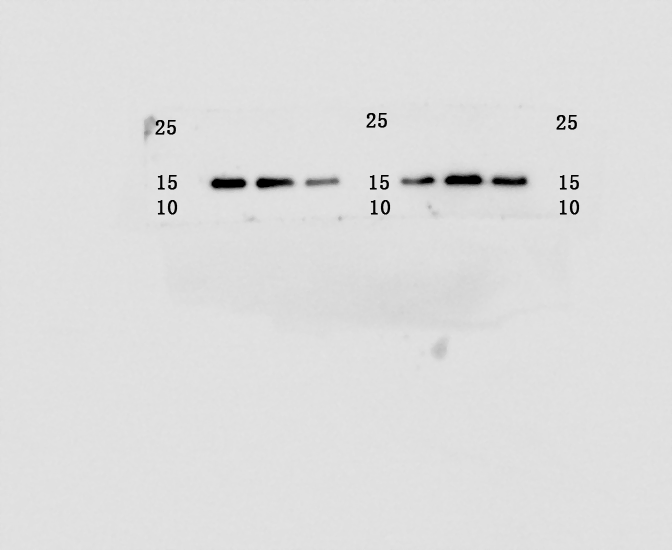


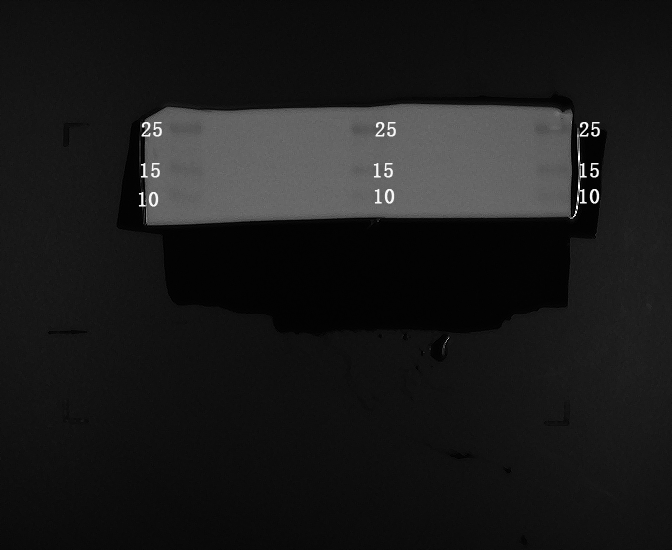


**Repeat 3 (Right)**

**BAX 23kDa**


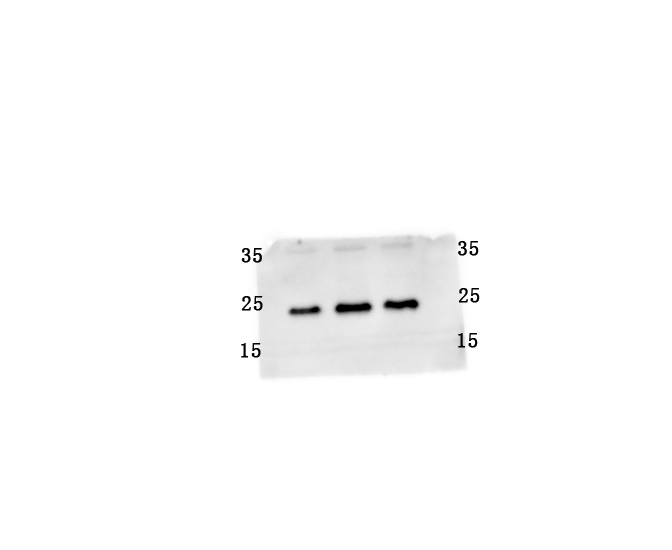


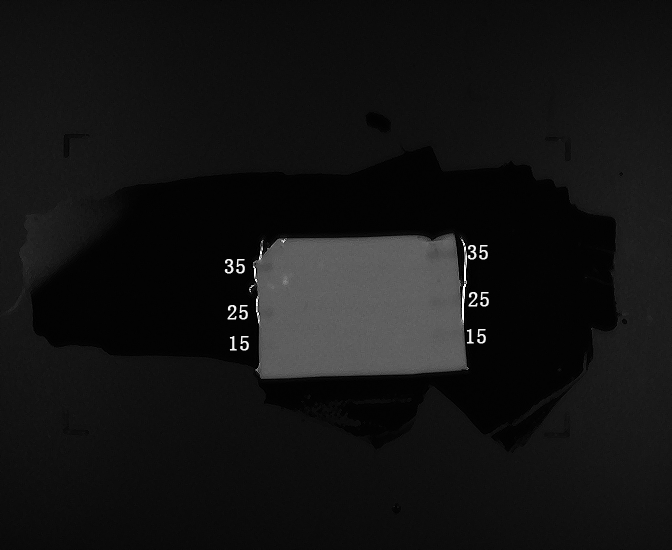


**Repeat 1**


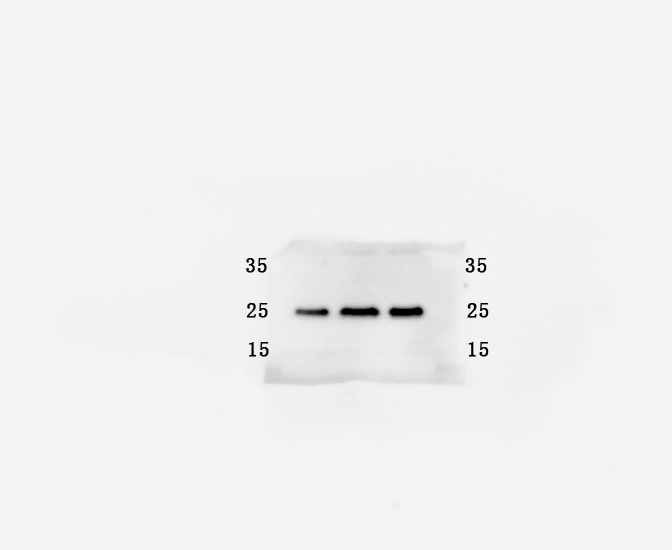


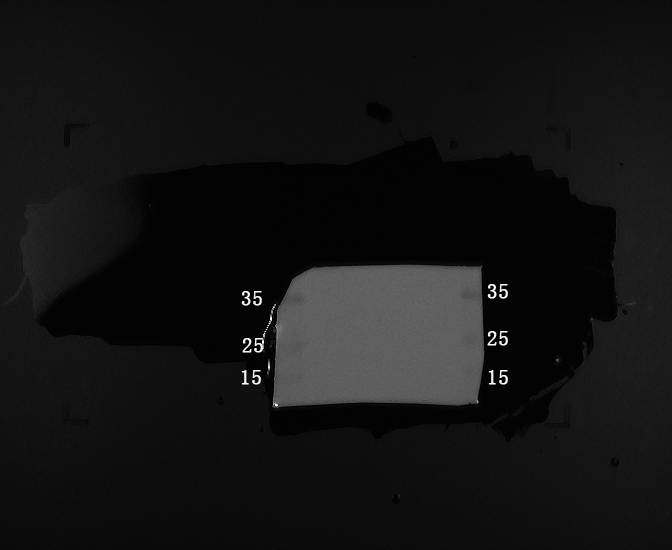


**Repeat 2**


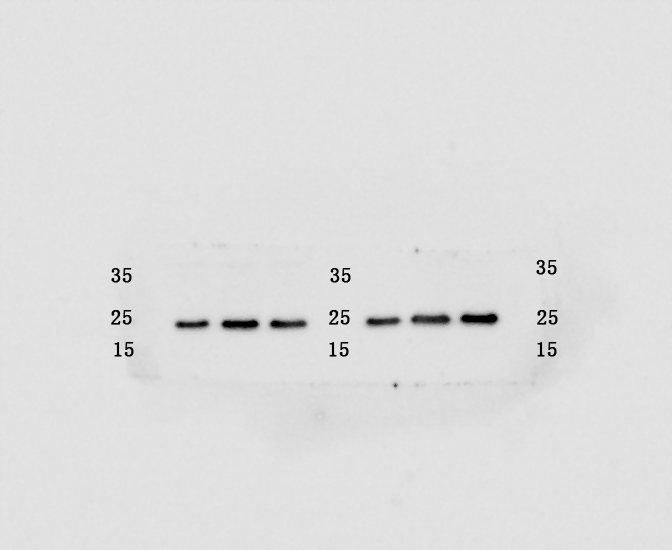


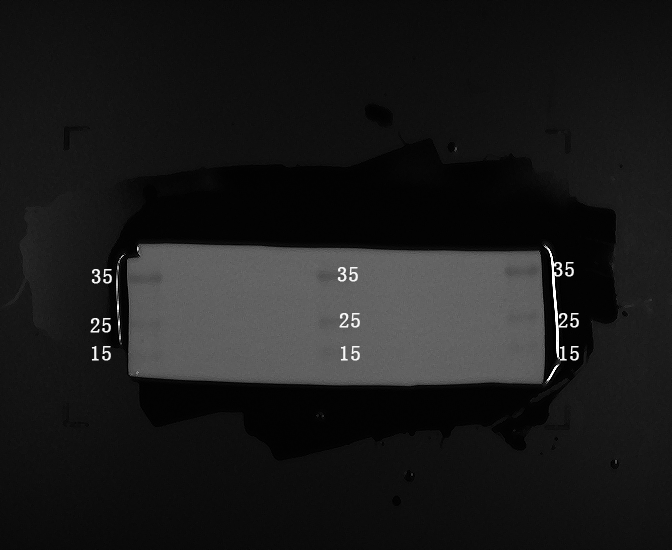


**Repeat 3(Left)**

**BCL-2 26kDa**


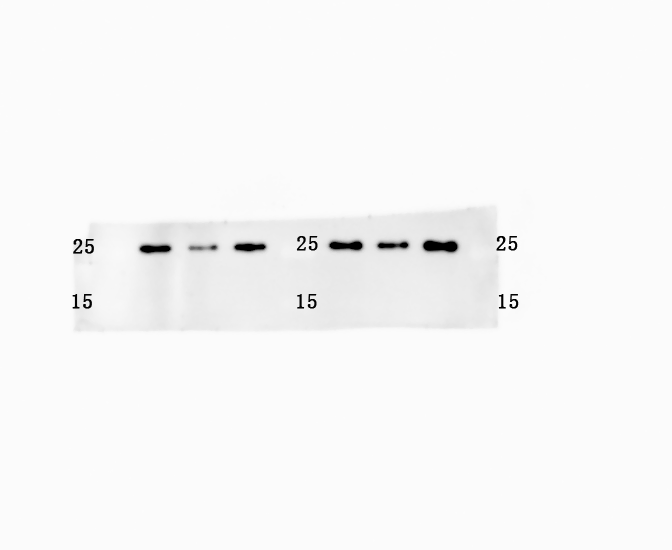


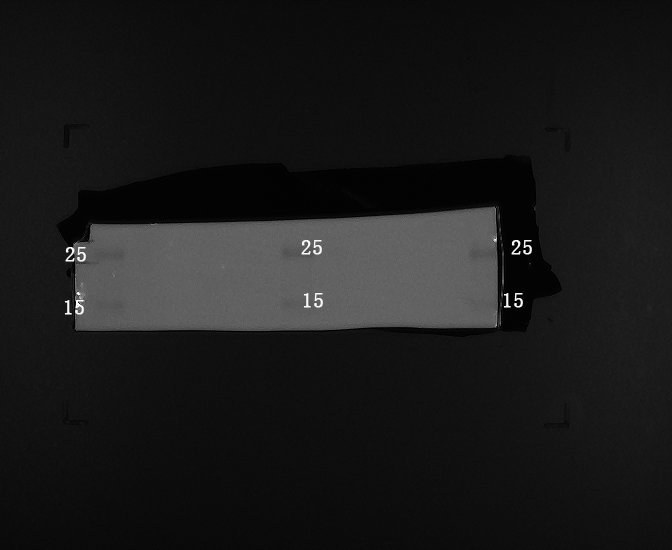


**Repeat 1 (Right)**


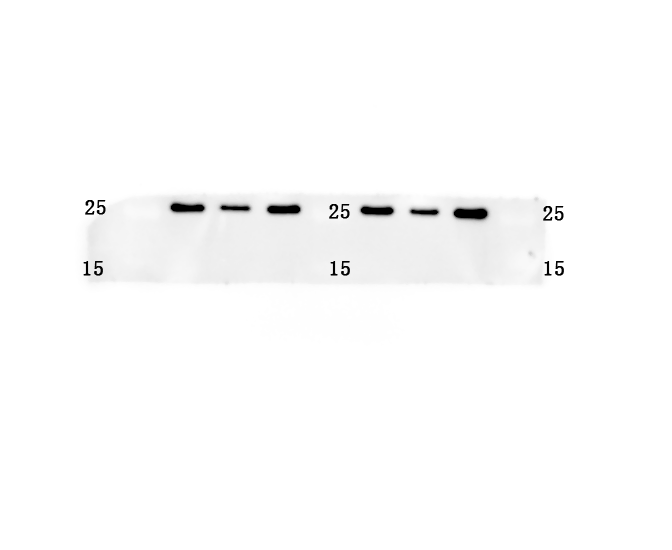


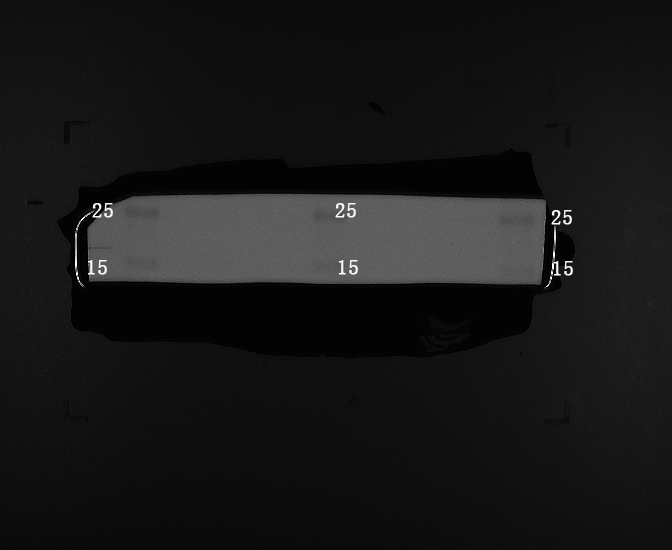


**Repeat 2+Repeat 3**

**(Fig.6A) β-actin 43kDa**

**
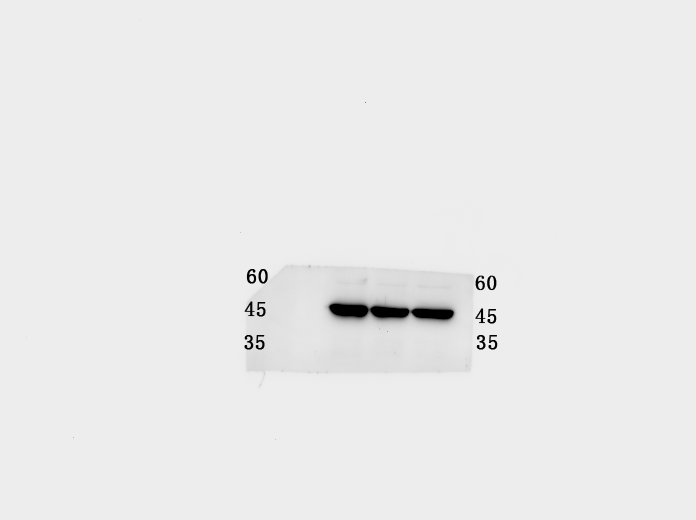
**

**
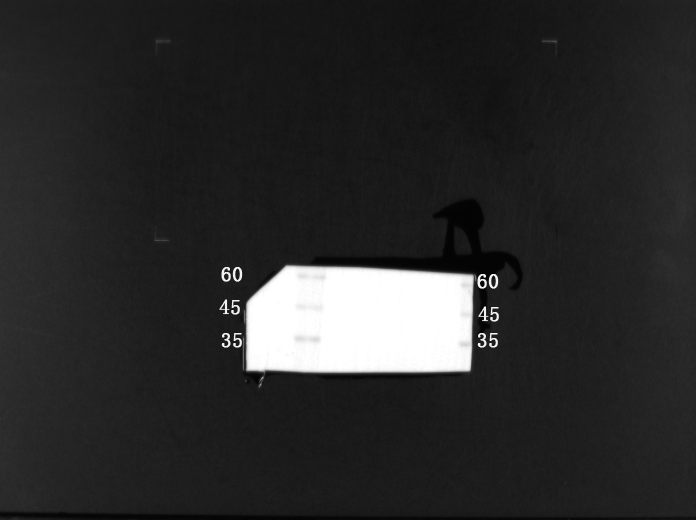
**

**Repeat 1**

**
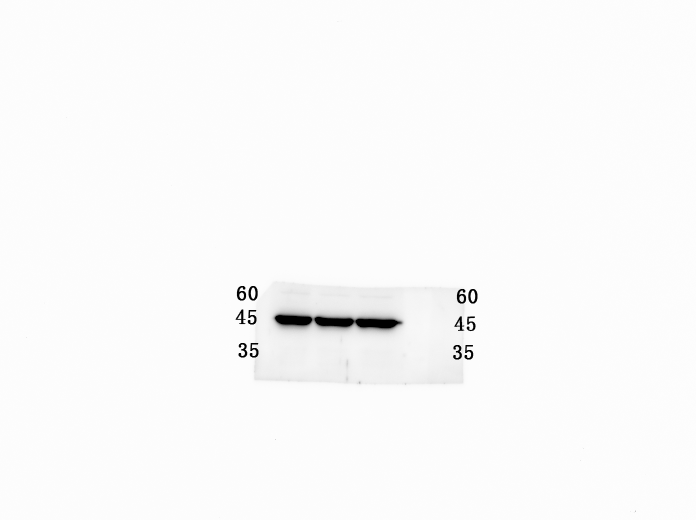
**

**
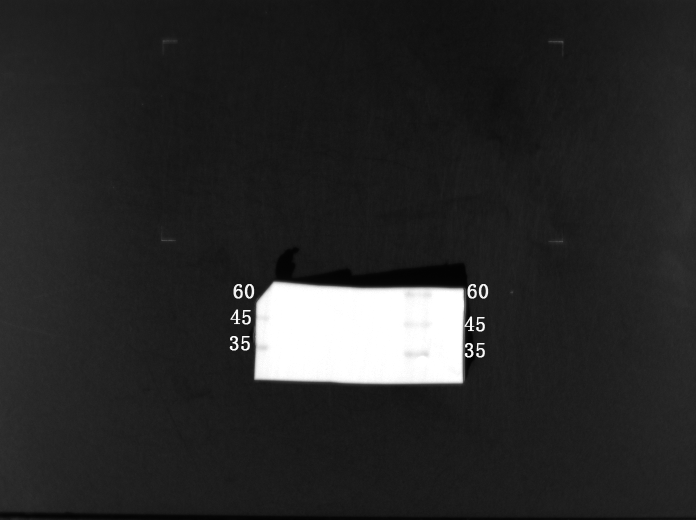
**

**Repeat 2**

**
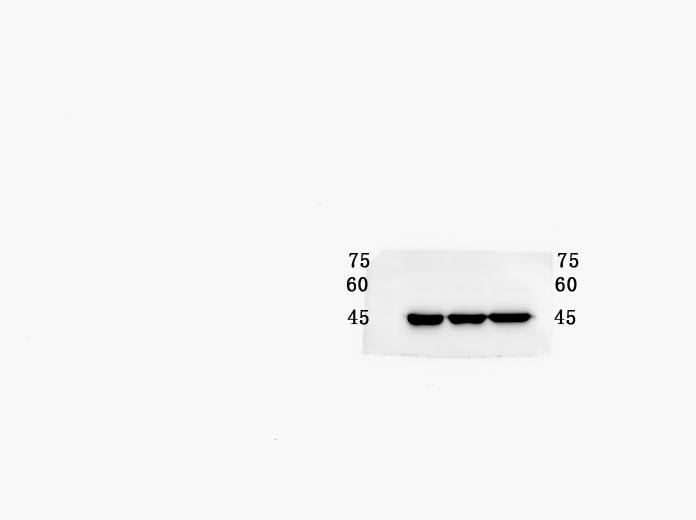
**

**
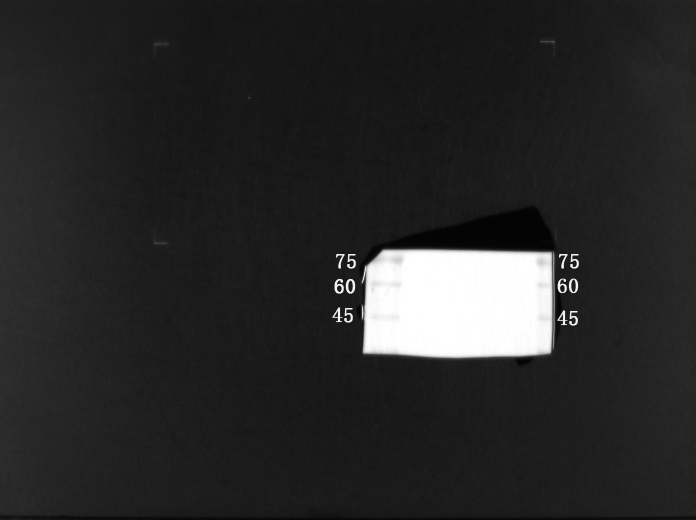
**

**Repeat 3**

**NLRP3 118kDa**

**
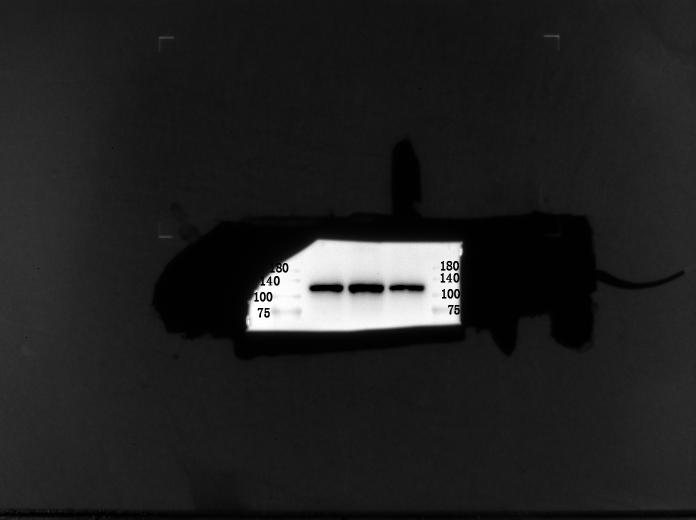
**


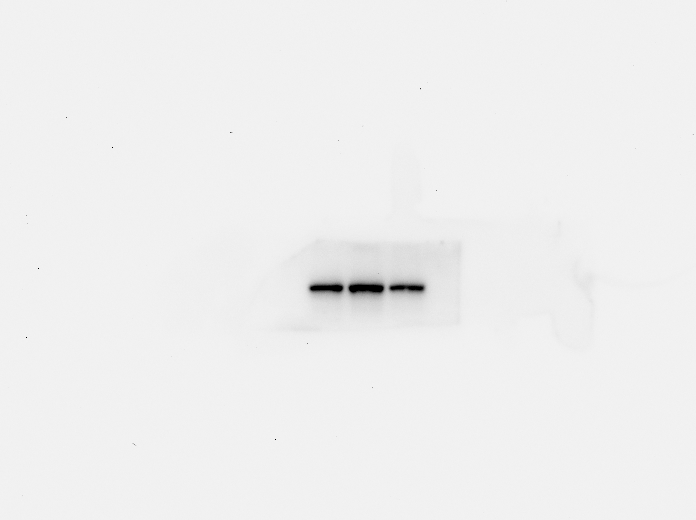


**Repeat 1**

**
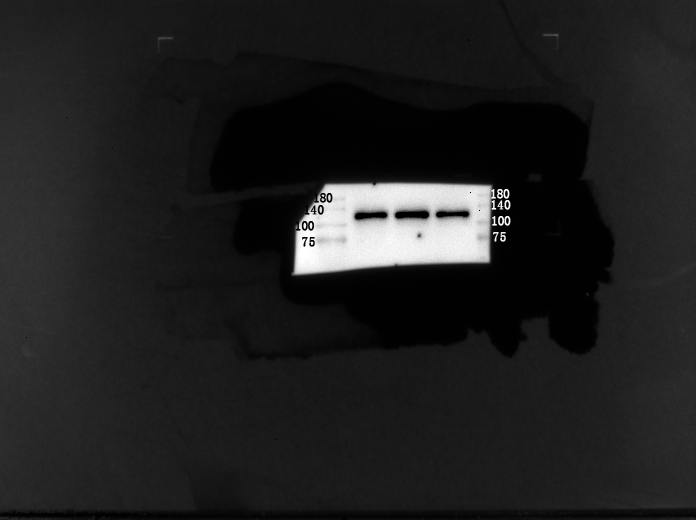
**


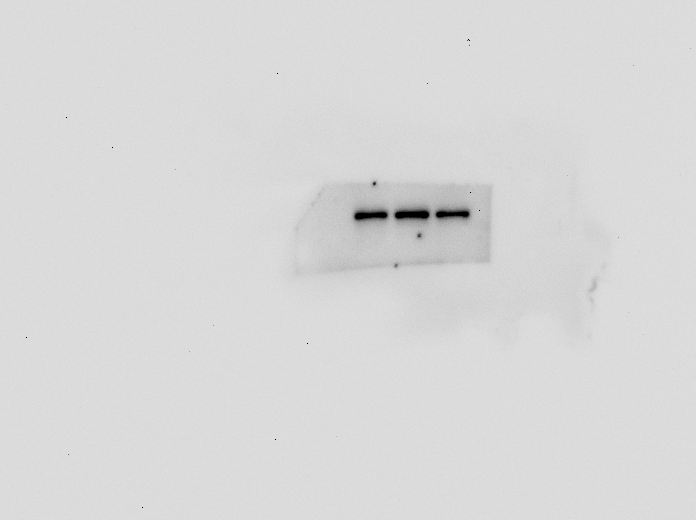


**Repeat 2**

**
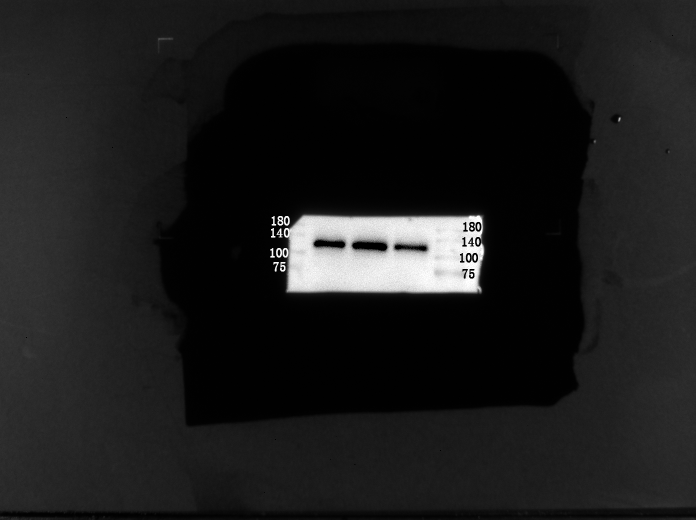
**


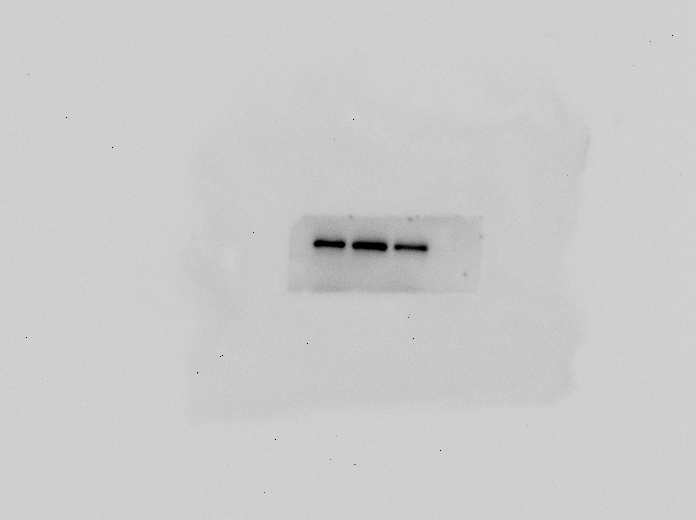


**Repeat 3**

**ASC 22kDa**

**
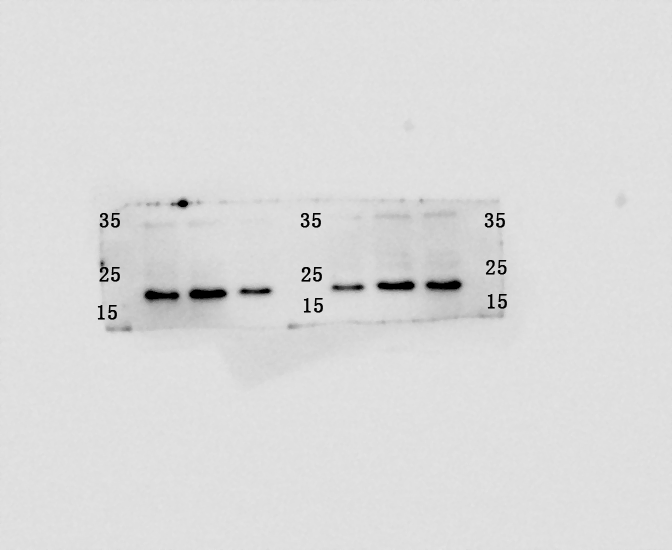
**

**
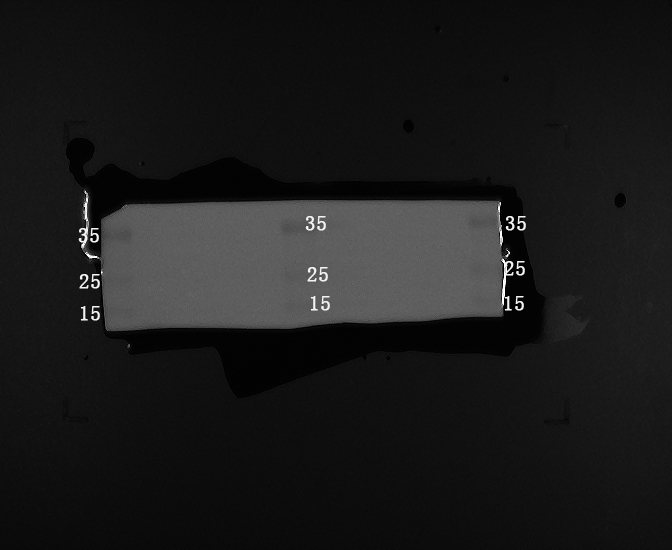
**

**Repeat 1** **(left)**

**
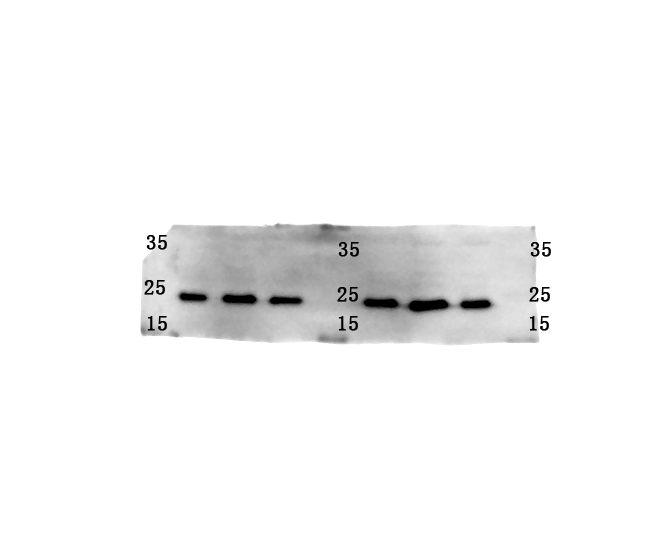
**

**
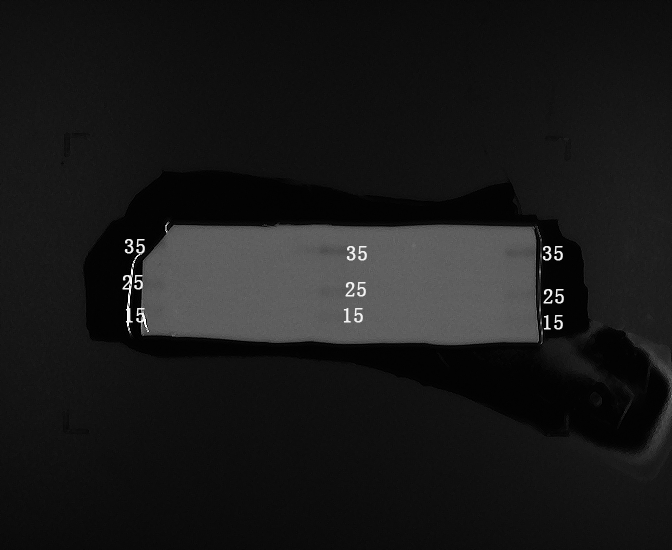
**

**Repeat 2+Repeat 3**

**cl-Caspase1 20kDa**

**
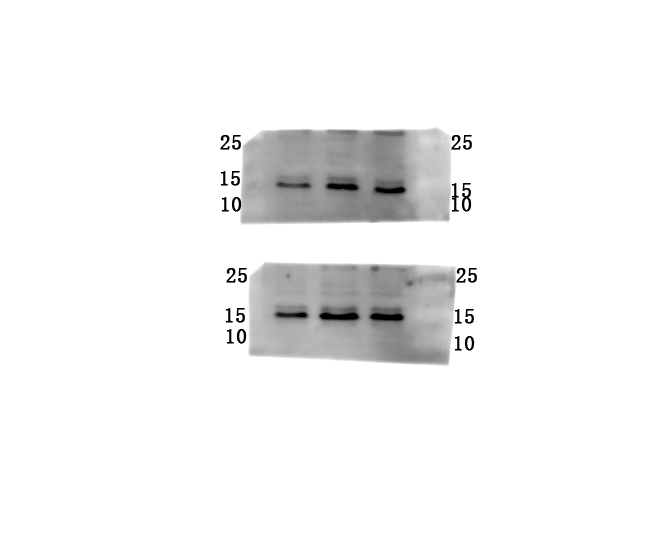
**

**
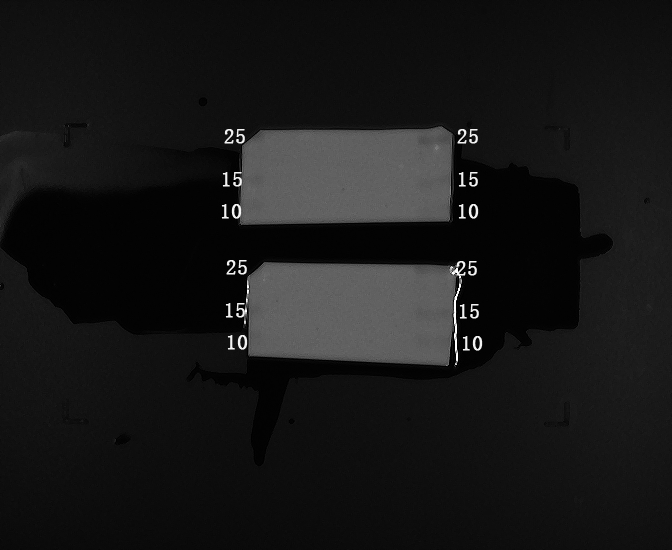
**

**Repeat 1+Repeat 2**

**
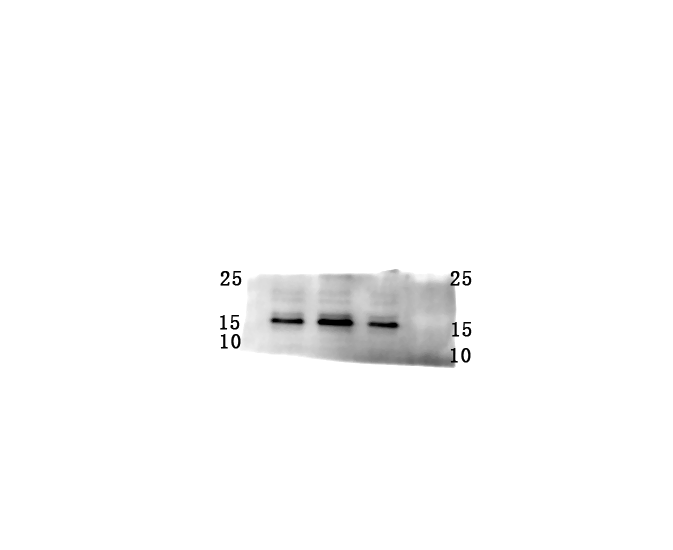
**

**
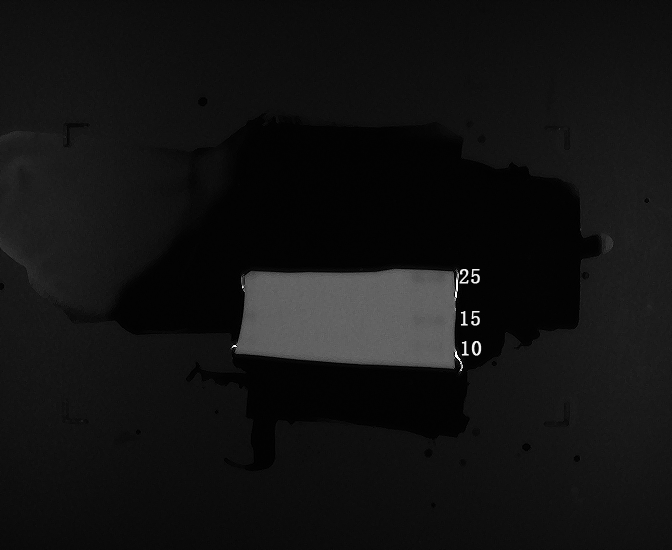
**

**Repeat 3**

**Caspase1 45kDa**

**
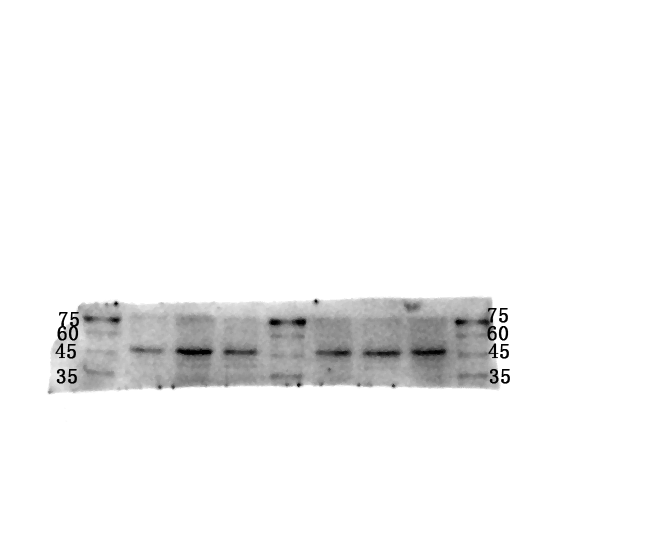
**

**
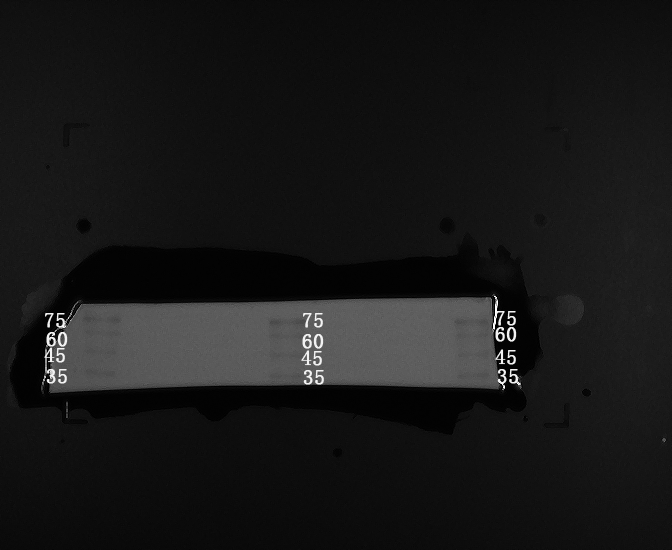
**

**Repeat 1****(left)**

**
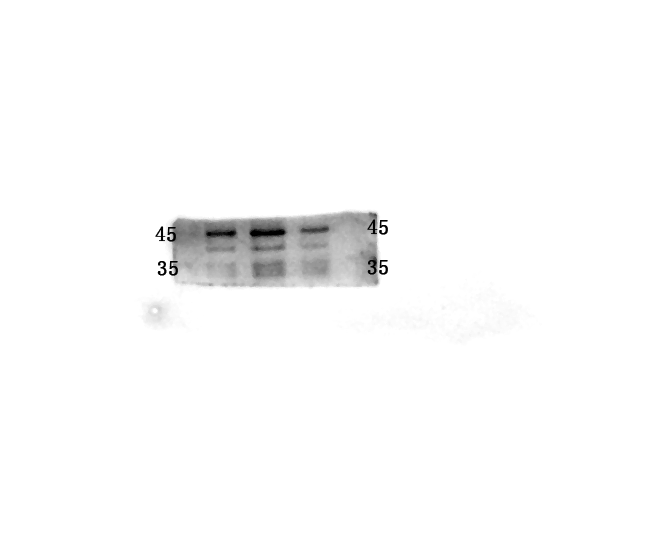
**

**
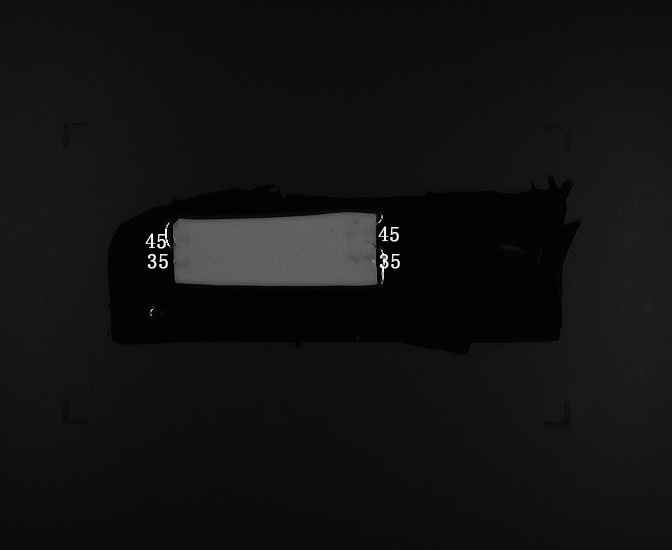
**

**Repeat 2**

**
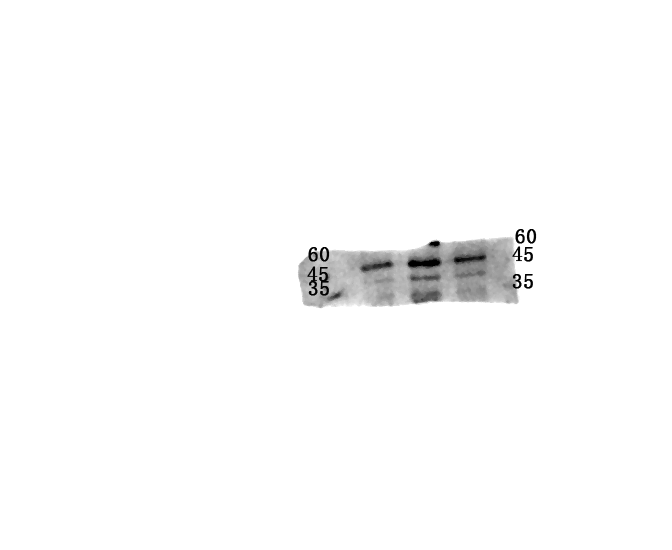
**

**
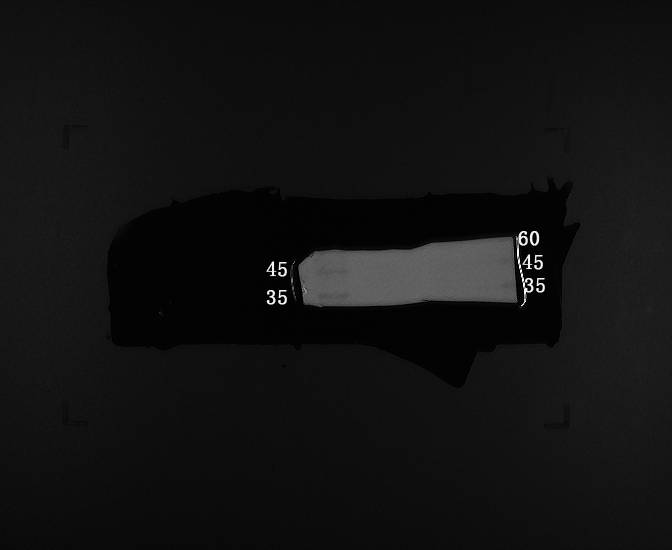
**

**Repeat 3**

**IL-18 22kDa**

**
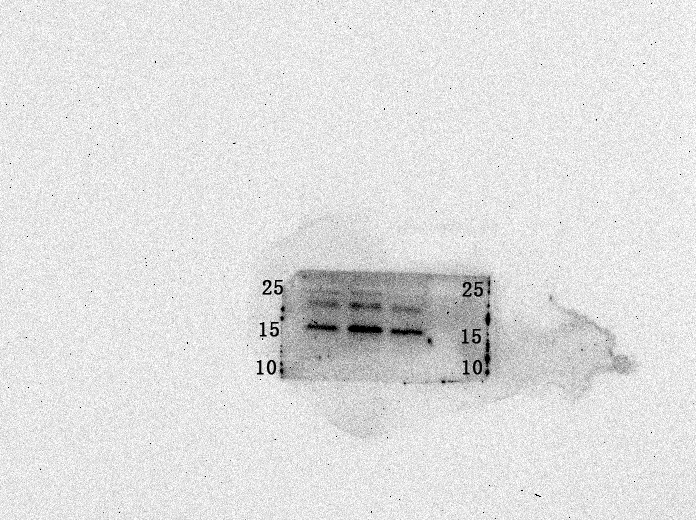
**

**
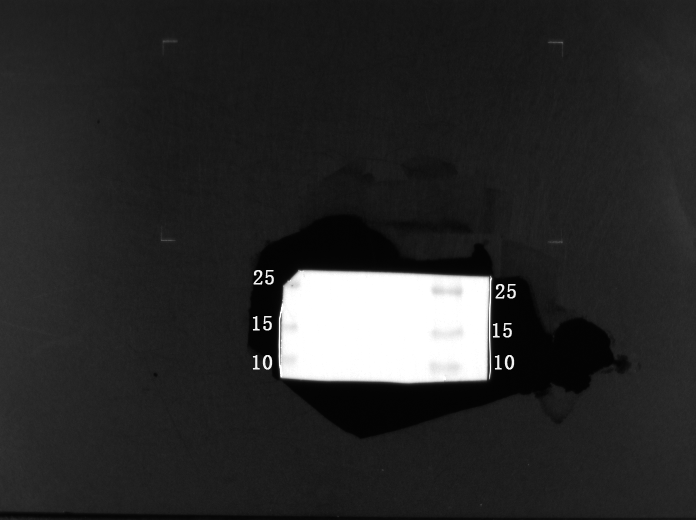
**

**Repeat 1**

**
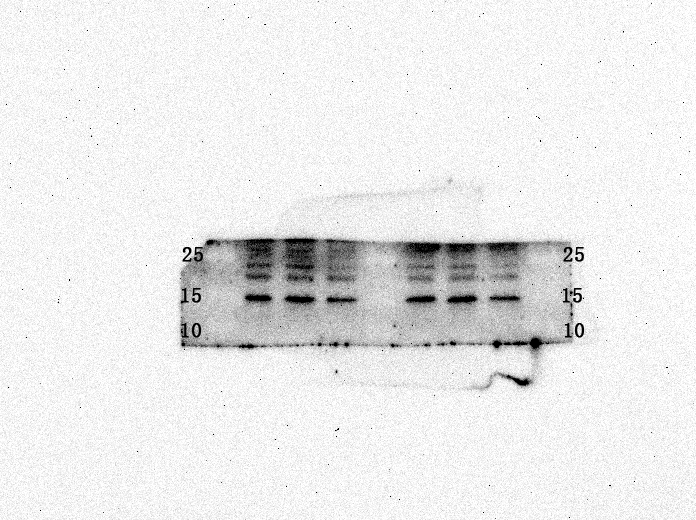
**

**
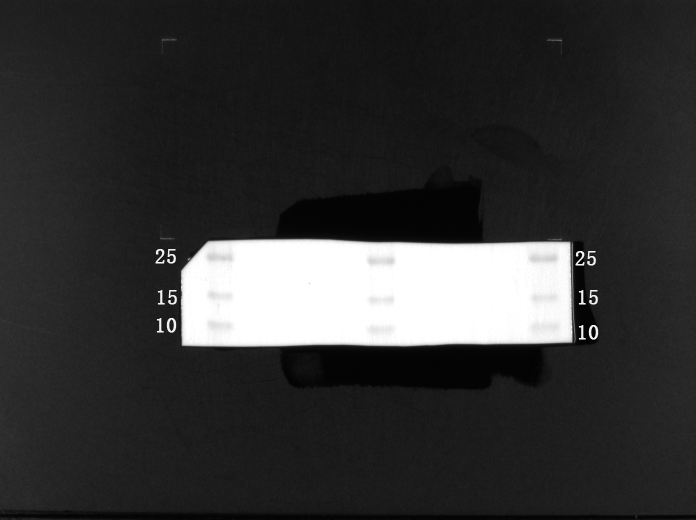
**

**Repeat 2(left)**

**
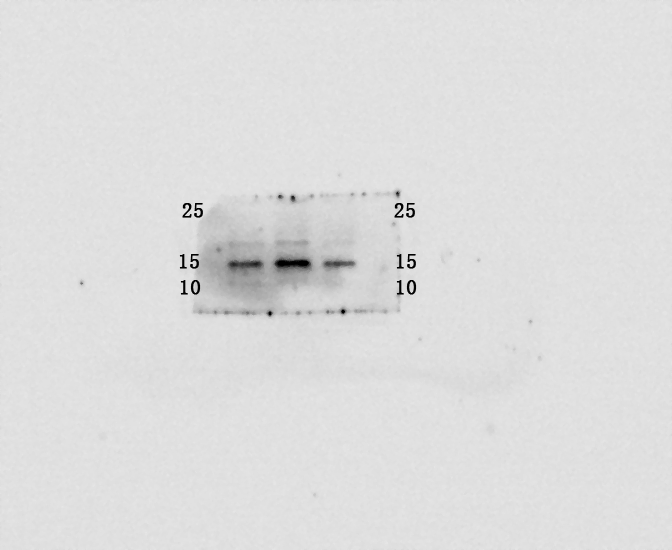
**

**
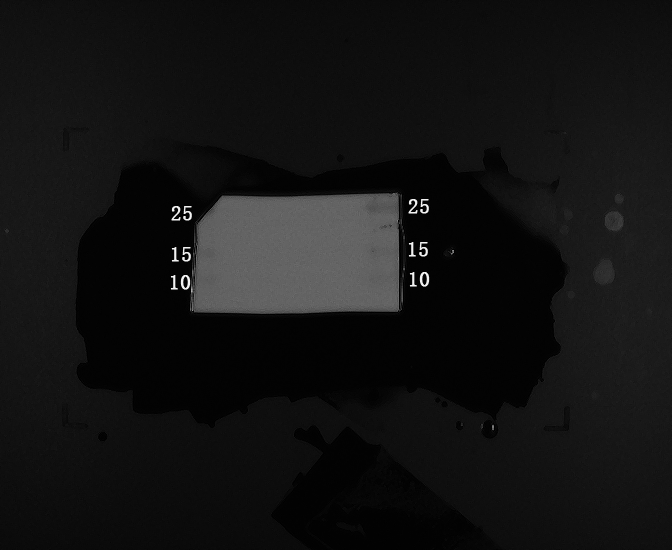
**

**Repeat 3**

**IL-** **1β 17kDa**

**
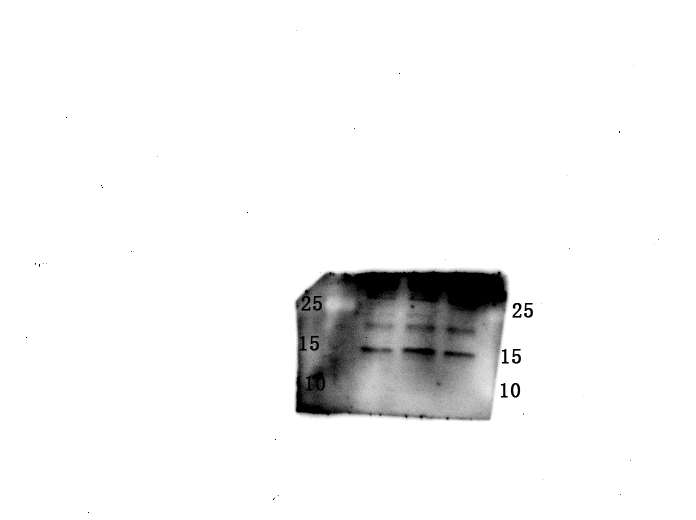
**

**
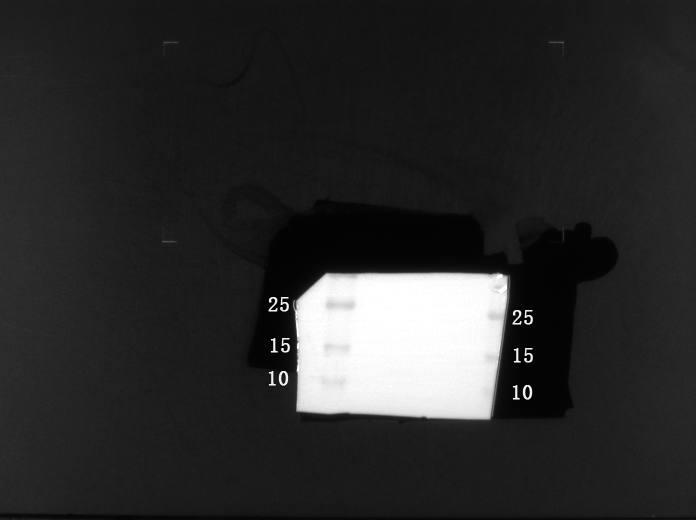
**

**Repeat 1**

**
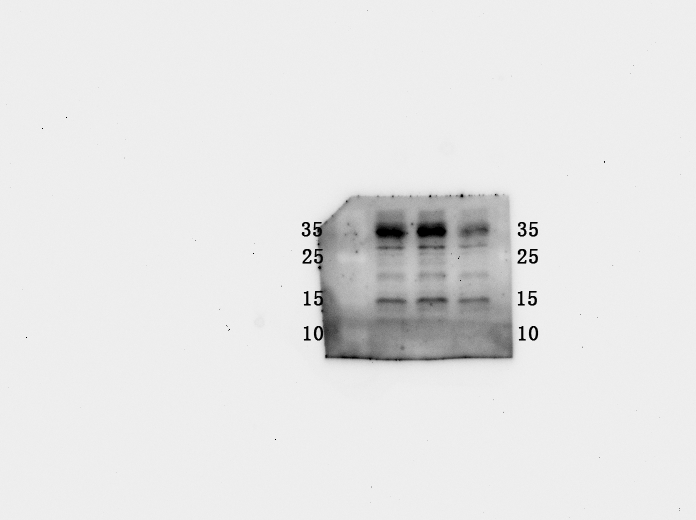
**

**
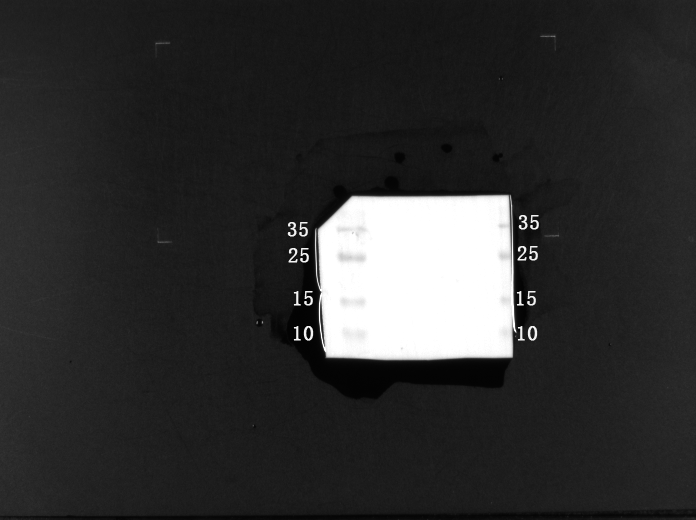
**

**Repeat 2**

**
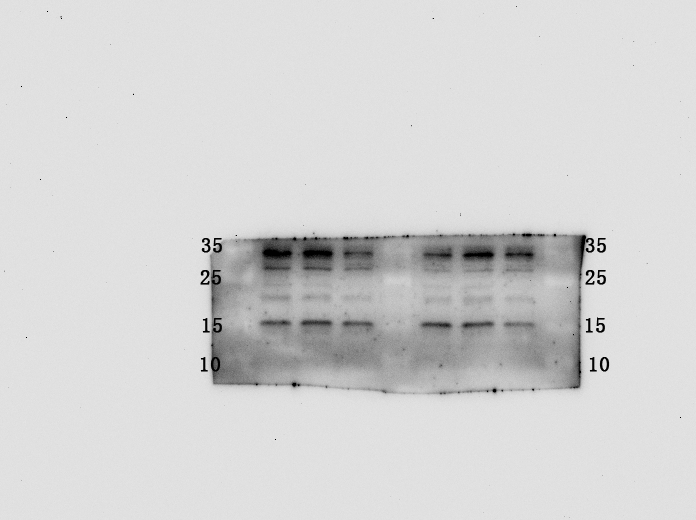
**

**
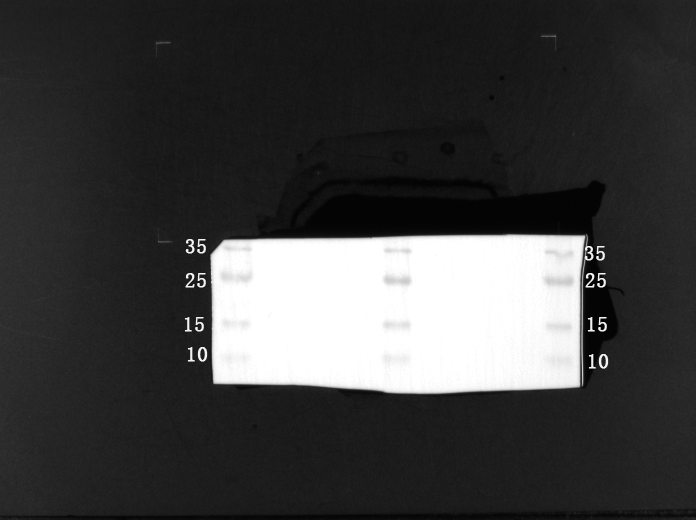
**

**Repeat 3(left)**

**GSDMD 53kDa**

**
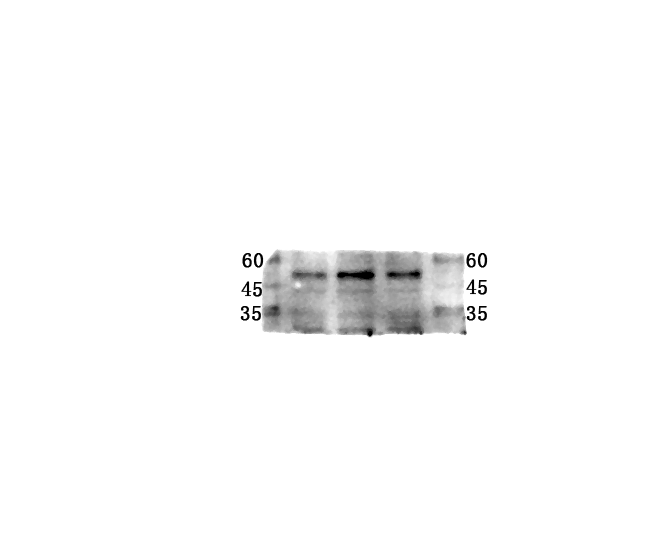
**

**
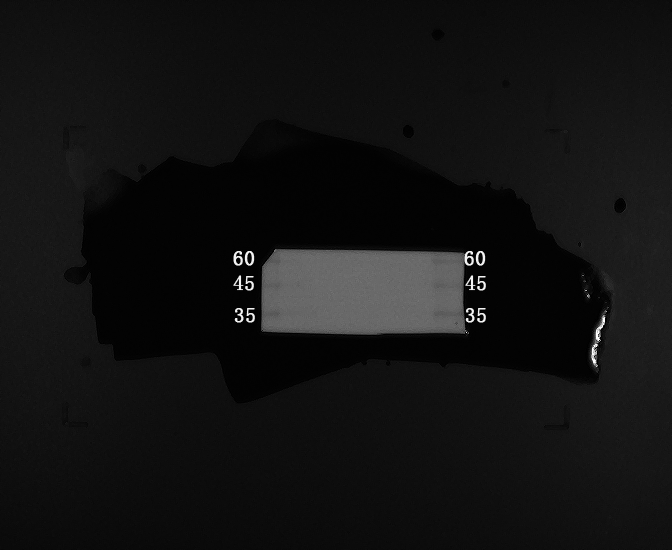
**

**Repeat 1**

**
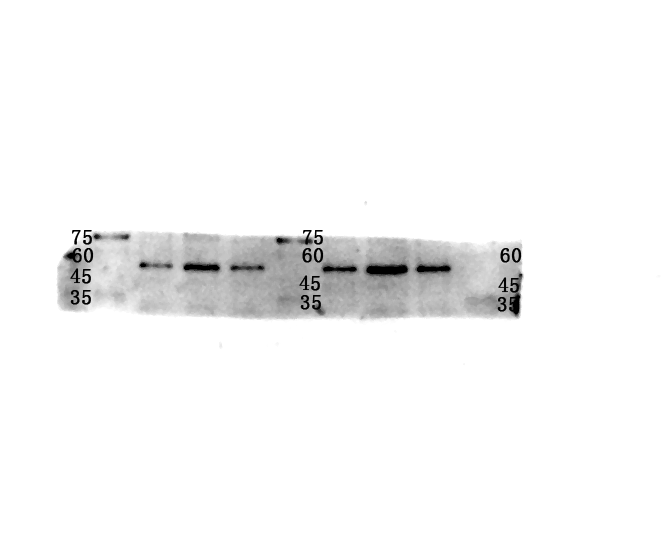
**

**
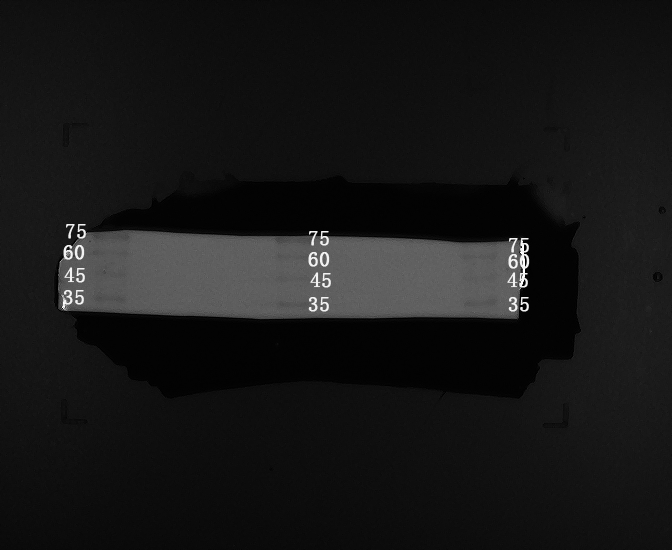
**

**Repeat 2+Repeat 3**

**(Fig.8A)β-actin 43kDa**

**
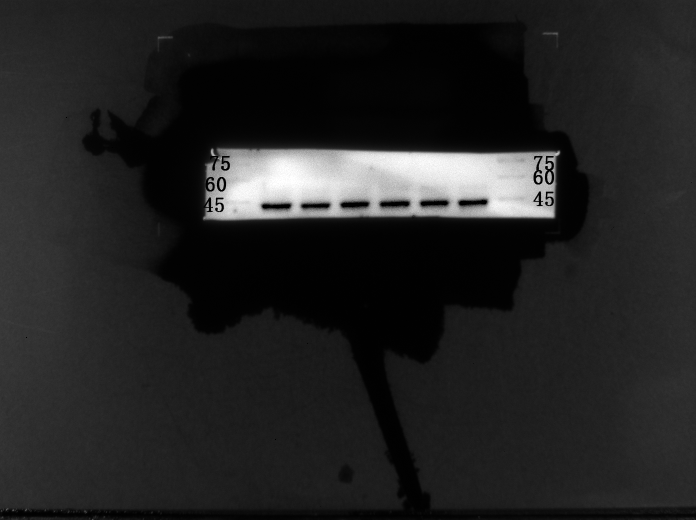
**


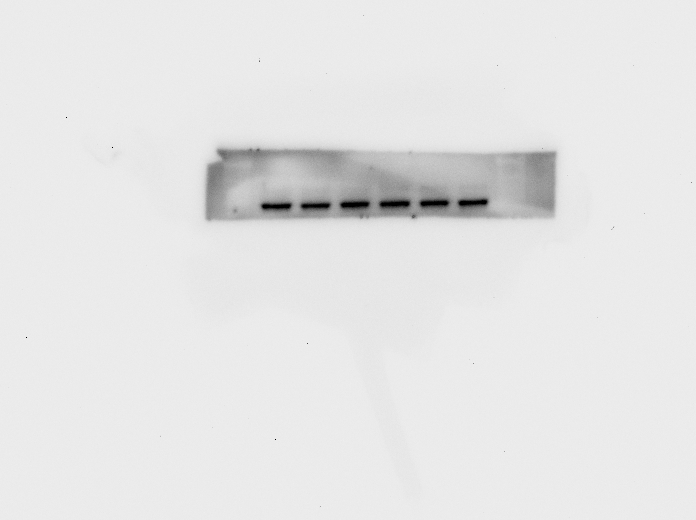


**Repeat 1**

**
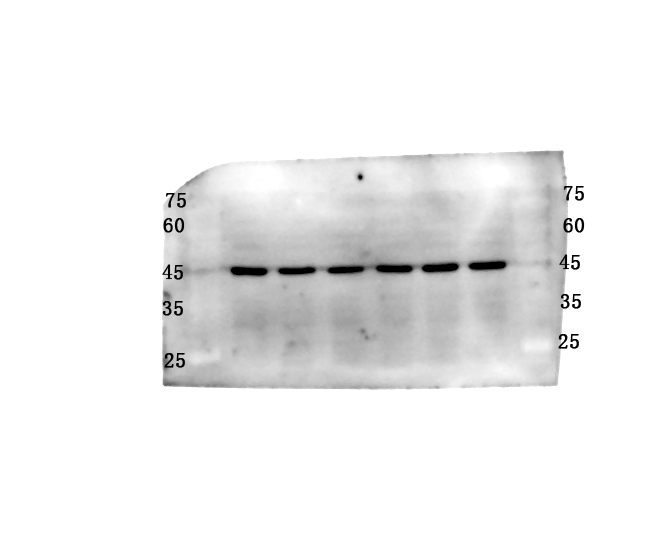
**

**
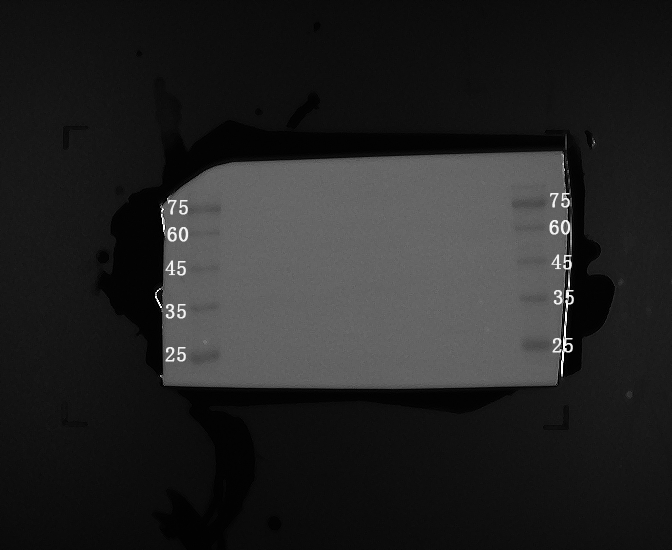
**

**Repeat 2**

**
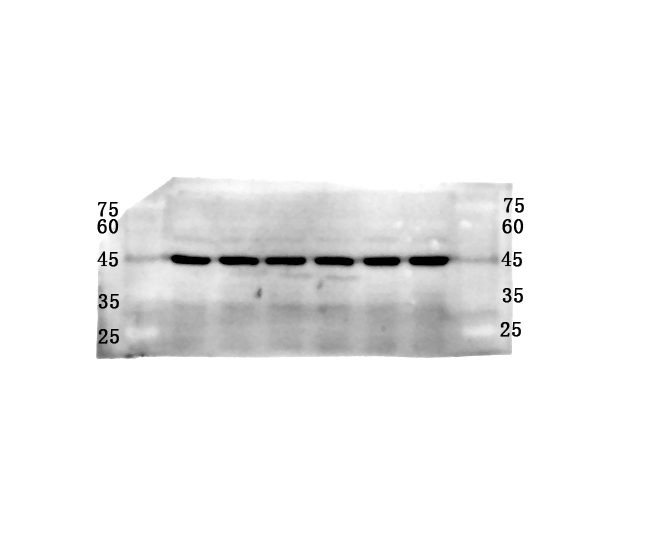
**

**
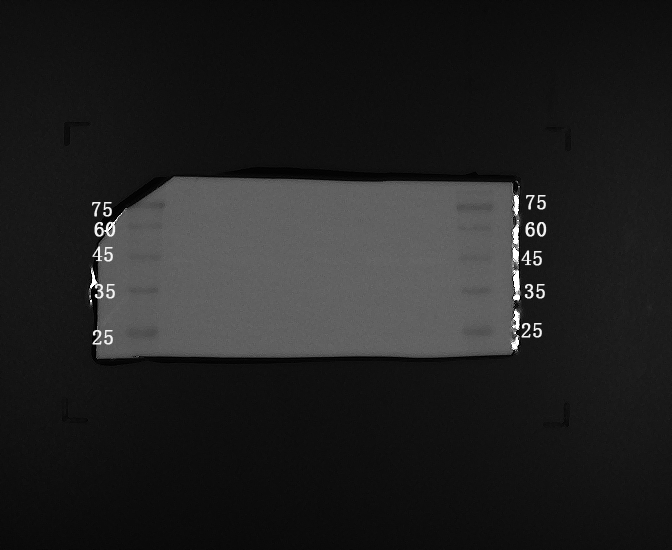
**

**Repeat 3**

**ASC 22kDa**

**
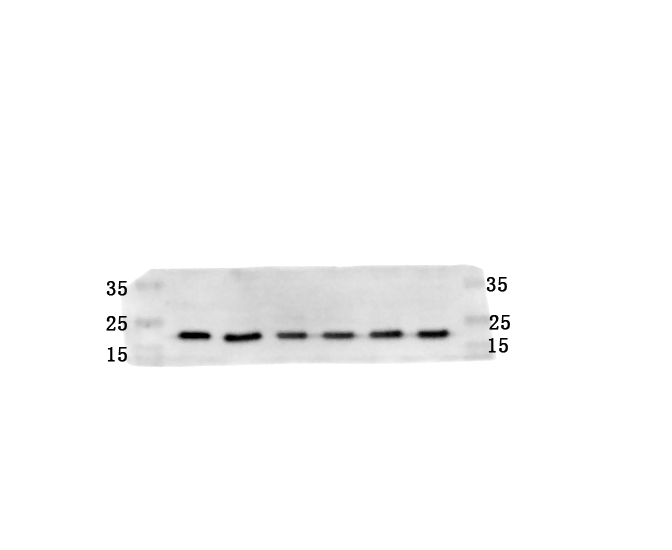
**

**
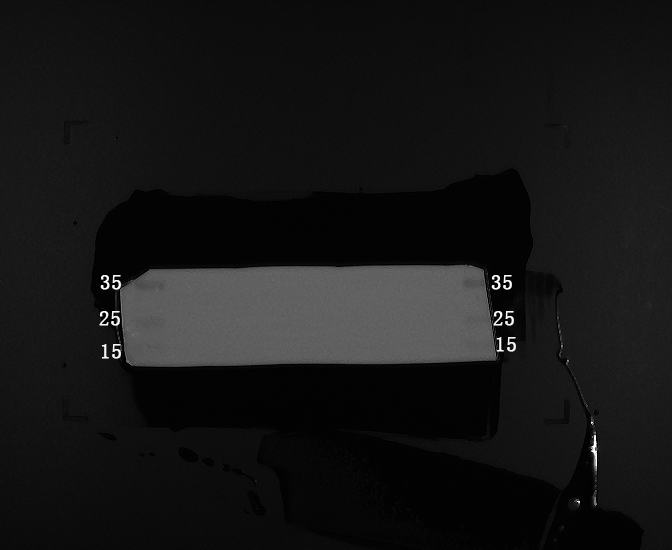
**

**Repeat 1**

**
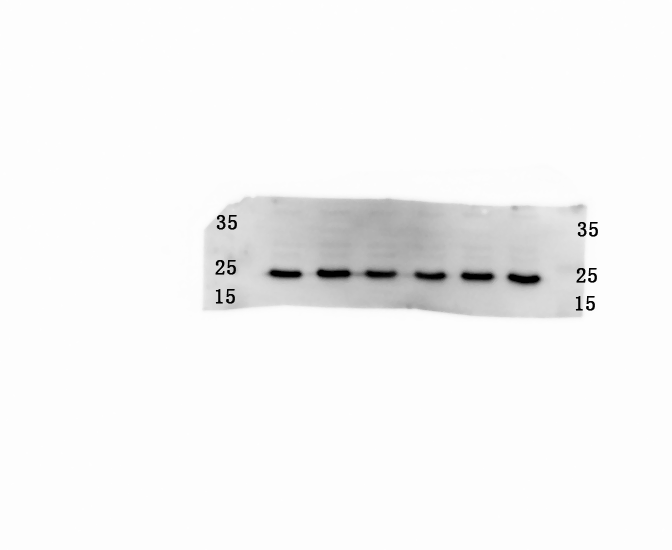
**

**
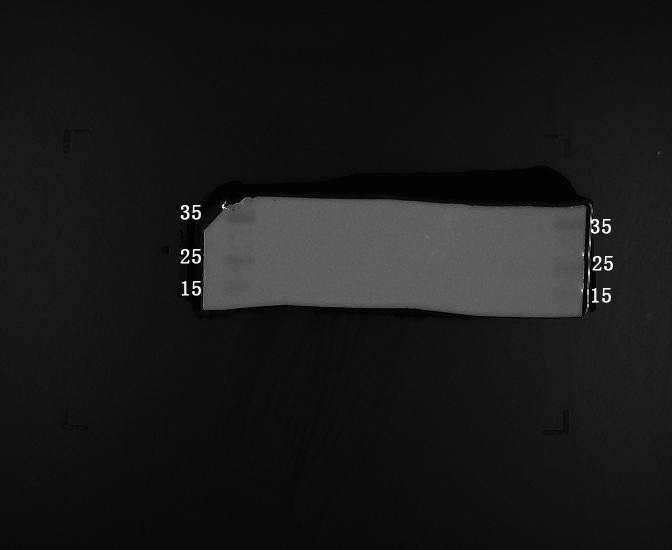
**

**Repeat 2**

**
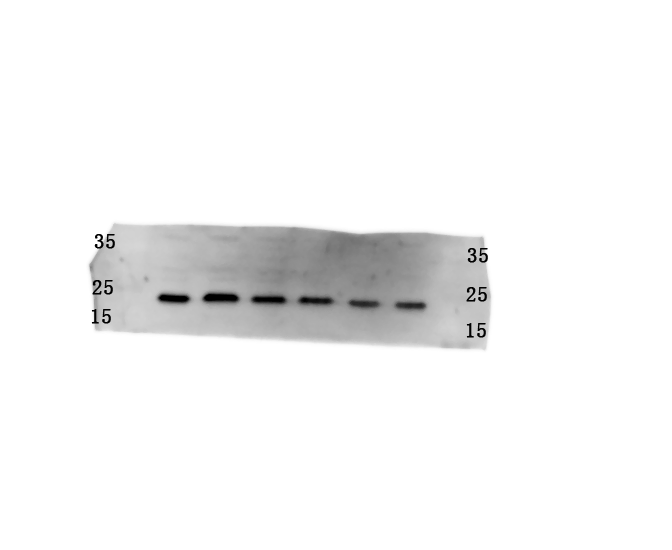
**

**
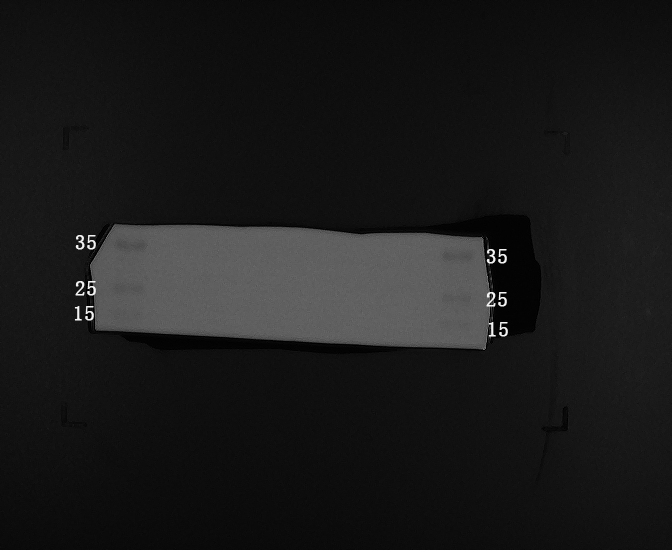
**

**Repeat 3**

**cl-Caspase1 20kDa**

**
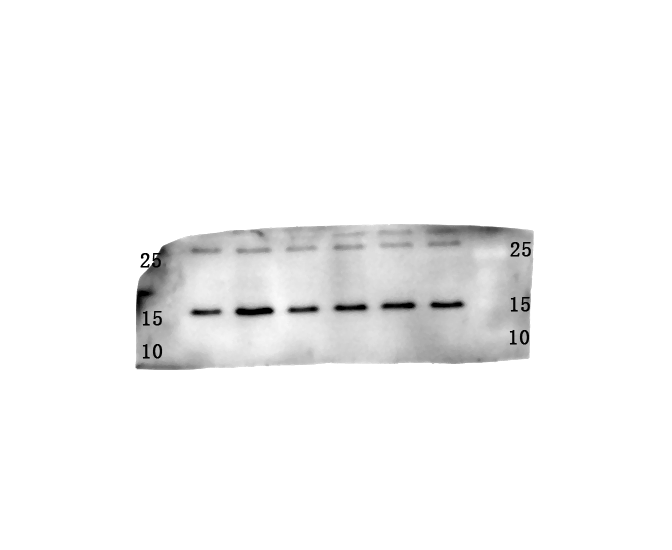
**

**
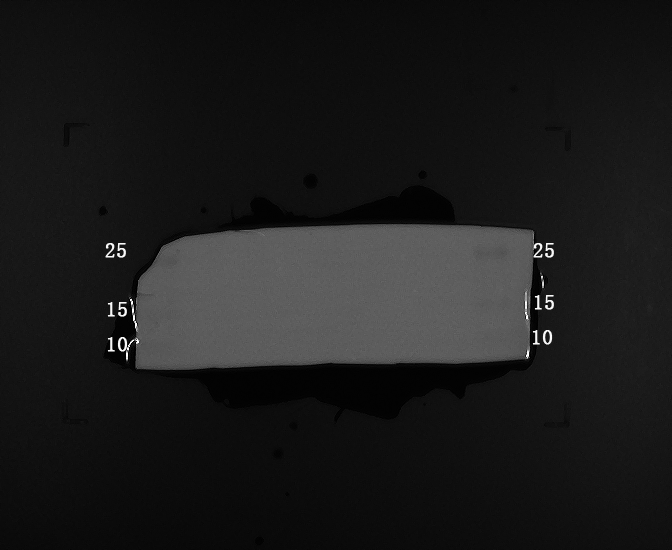
**

**Repeat 1**

**
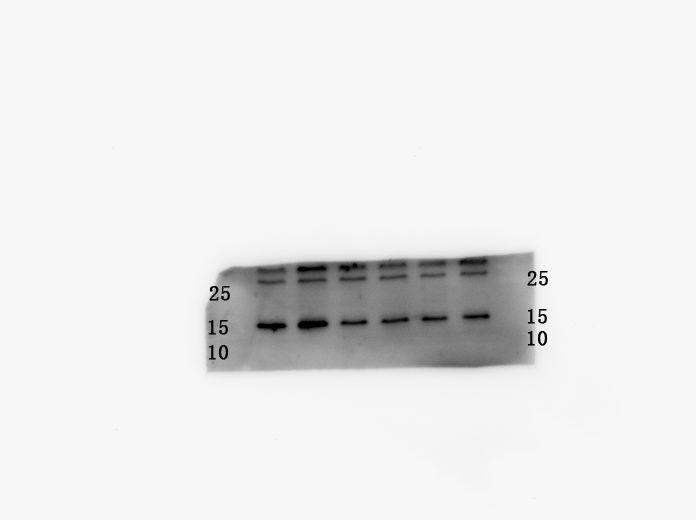
**

**
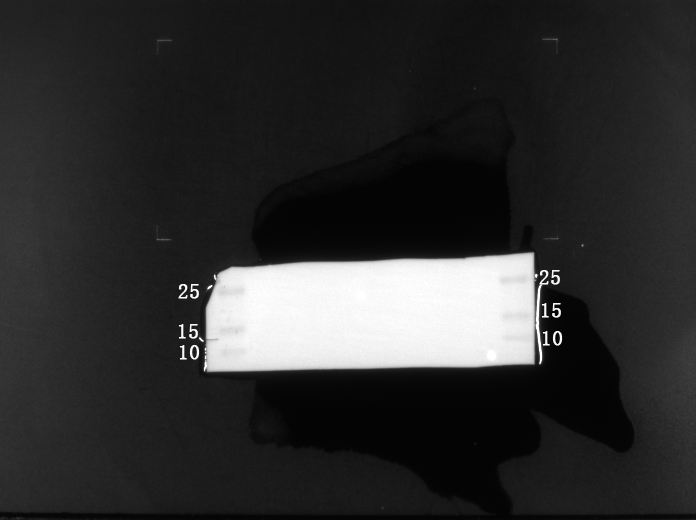
**

**Repeat 2**

**
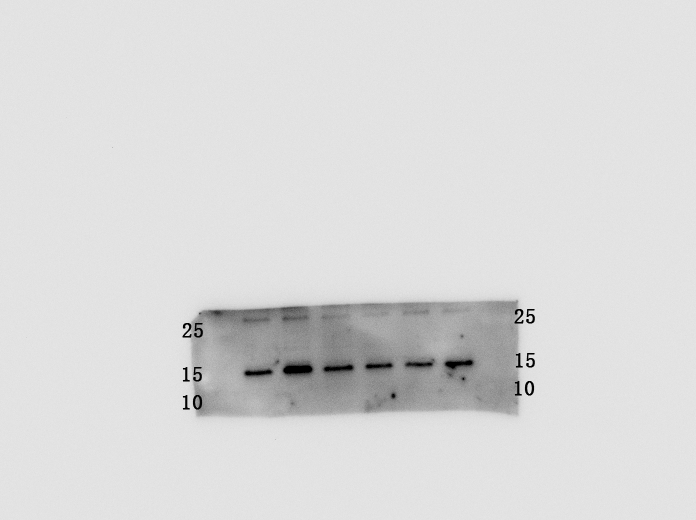
**

**
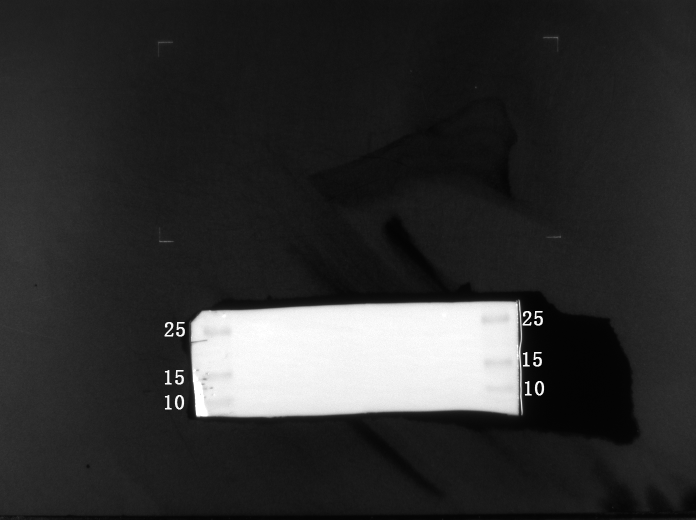
**

**Repeat 3**

**Caspase1 45kDa**

**
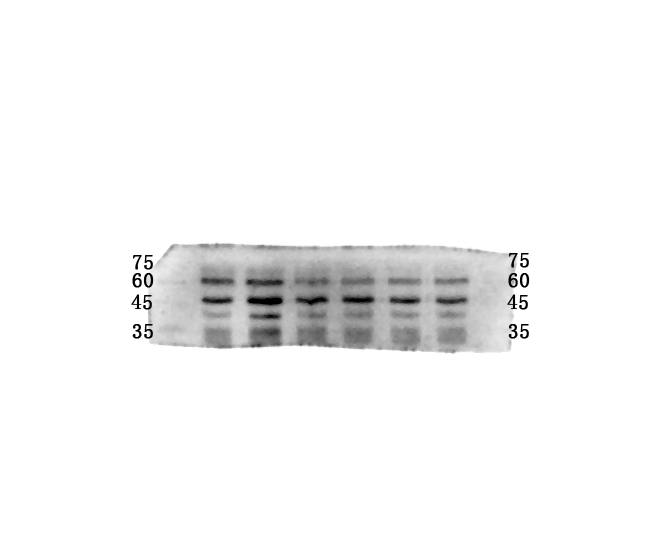
**

**
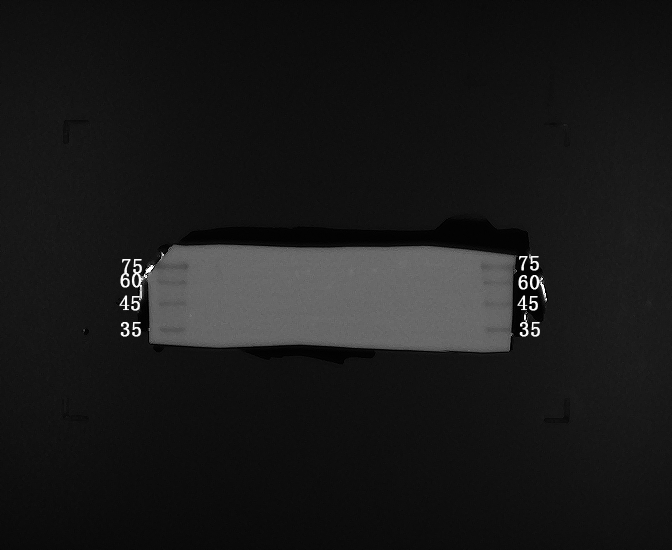
**

**Repeat 1**

**
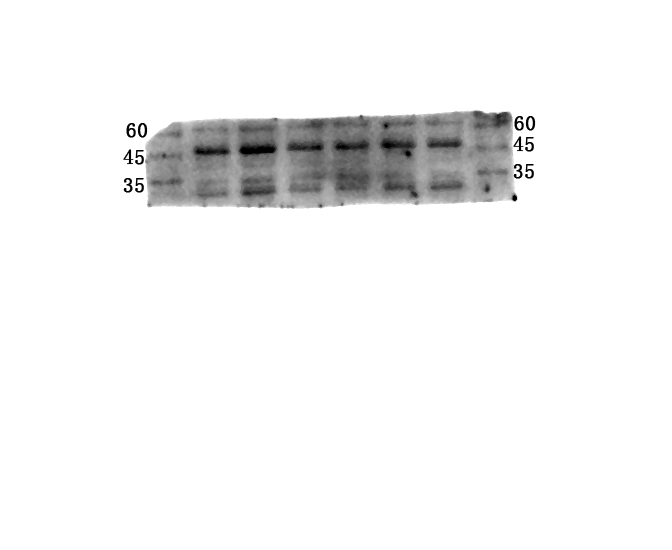
**

**
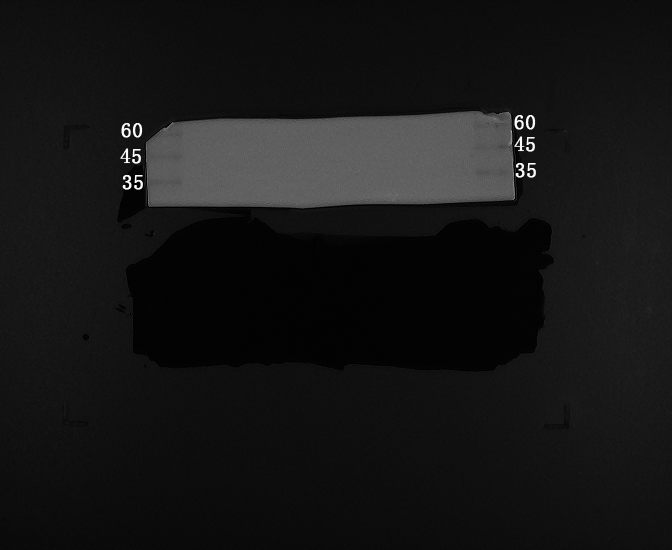
**

**Repeat 2**

**
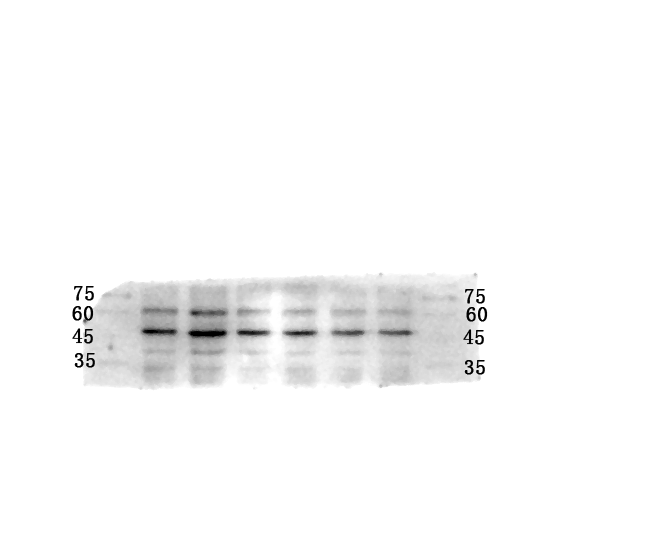
**

**
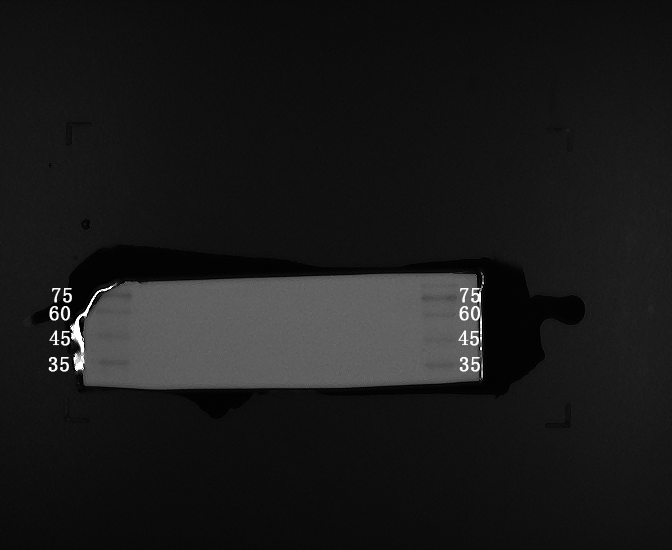
**

**Repeat 3**

**IL-18 22kDa**

**
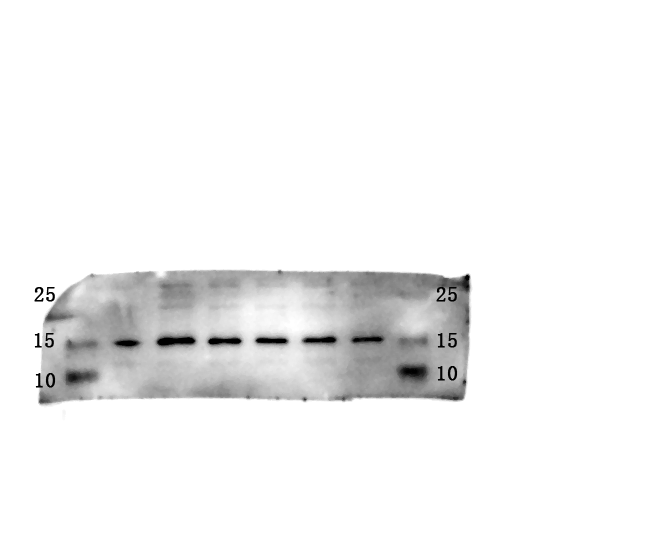
**

**
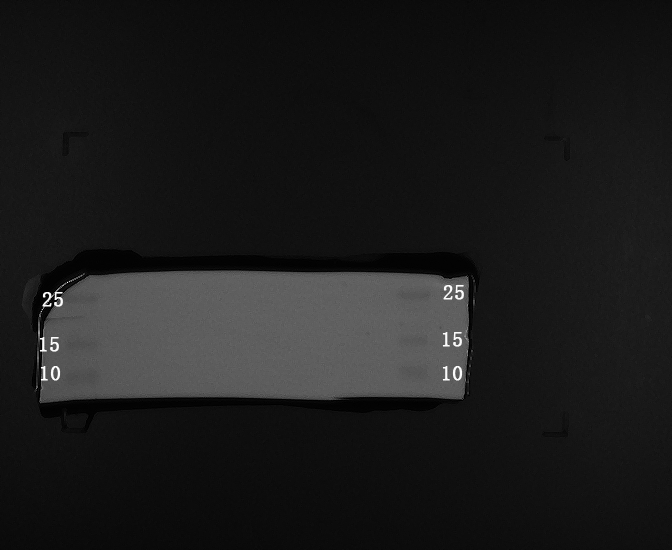
**

**Repeat 1**

**
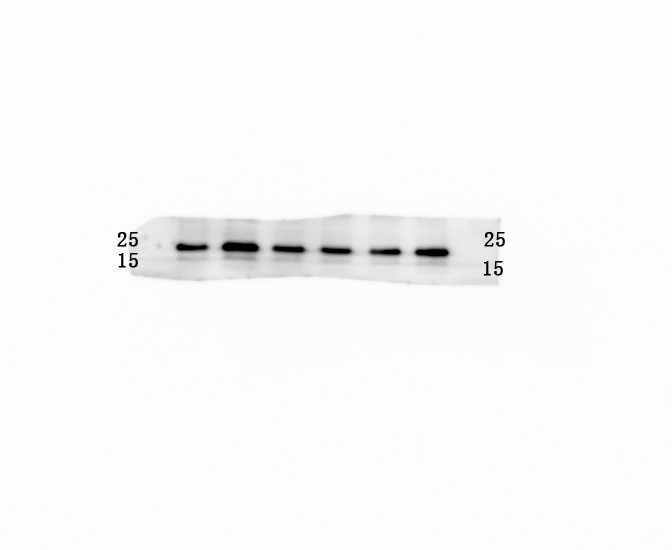
**

**
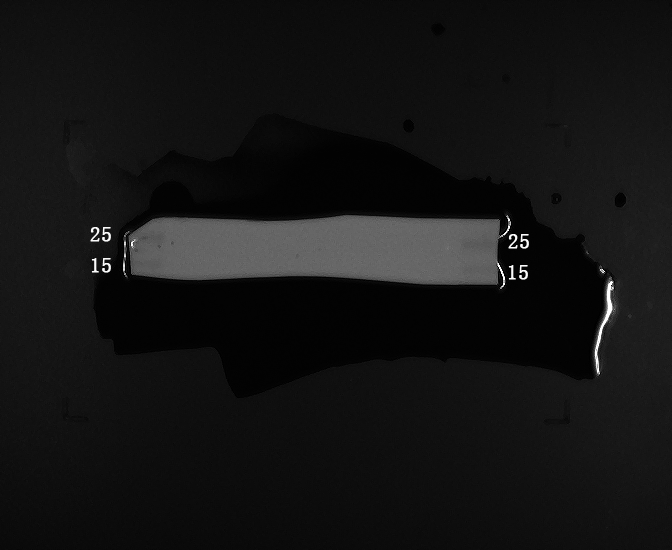
**

**Repeat 2**

**
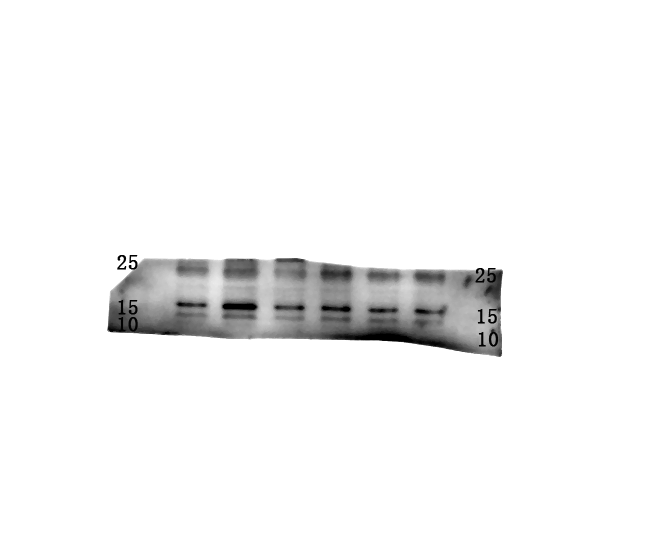
**

**
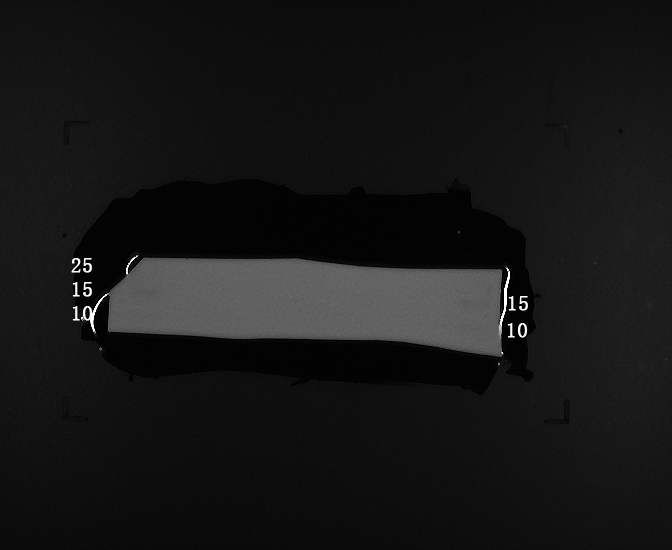
**

**Repeat 3**

**IL-** **1β 17kDa**

**
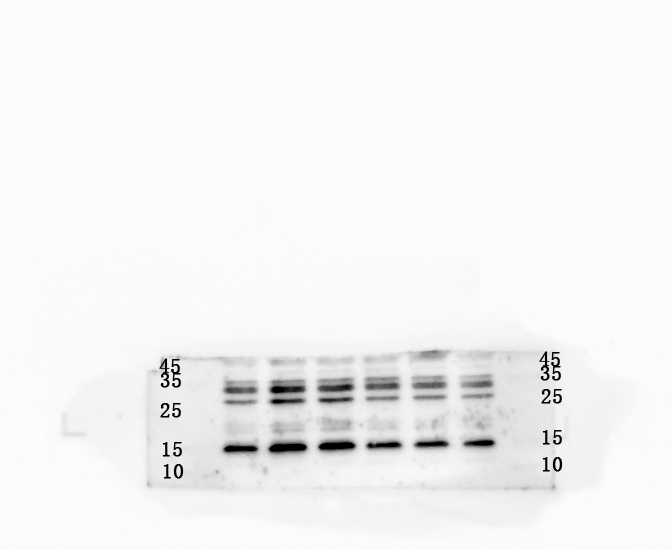
**

**
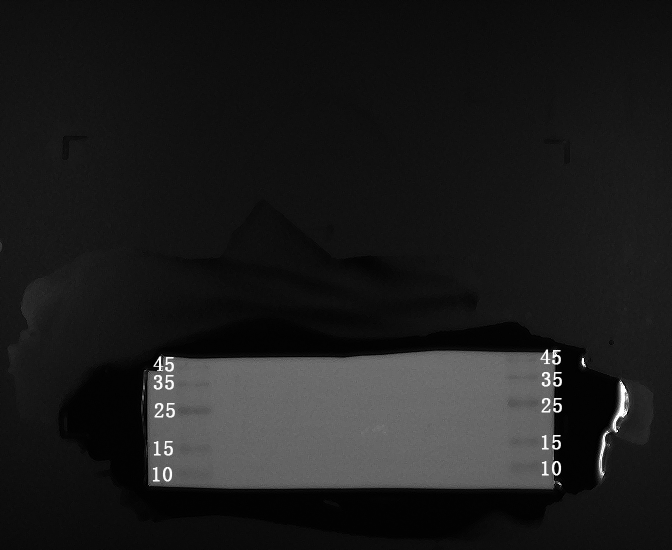
**

**Repeat 1**

**
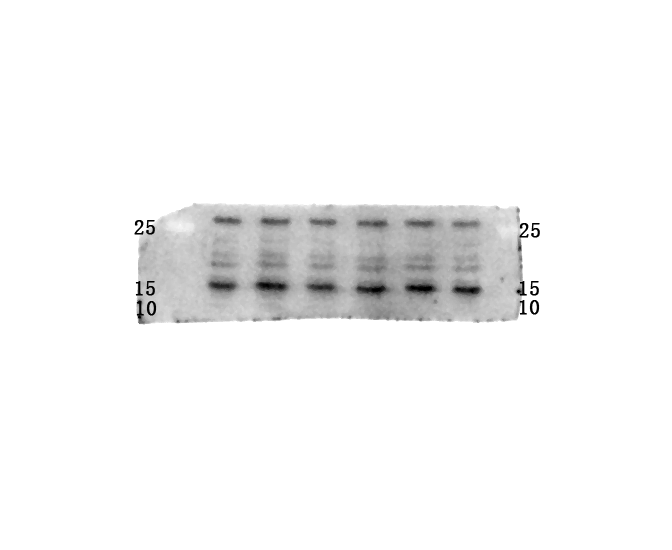
**

**
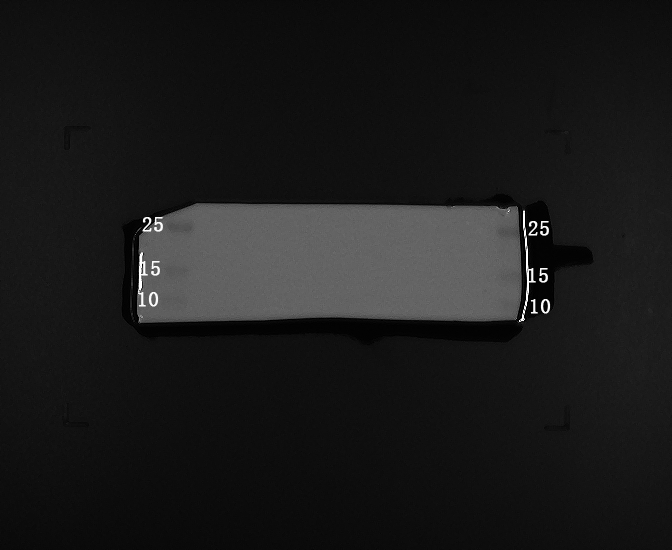
**

**Repeat 2**

**
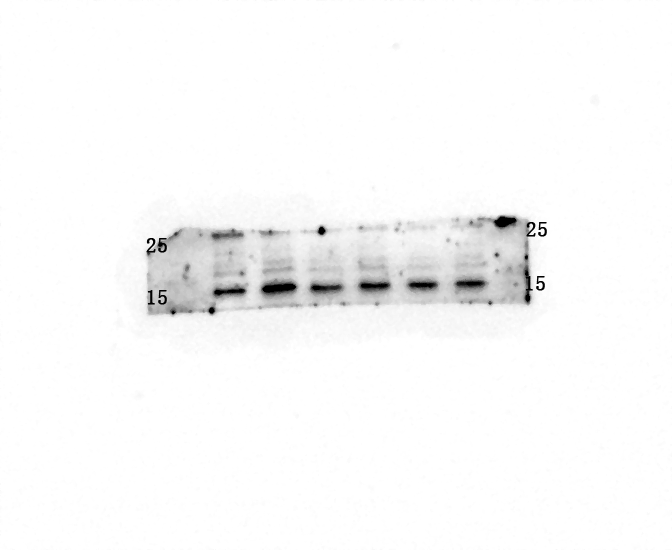
**

**
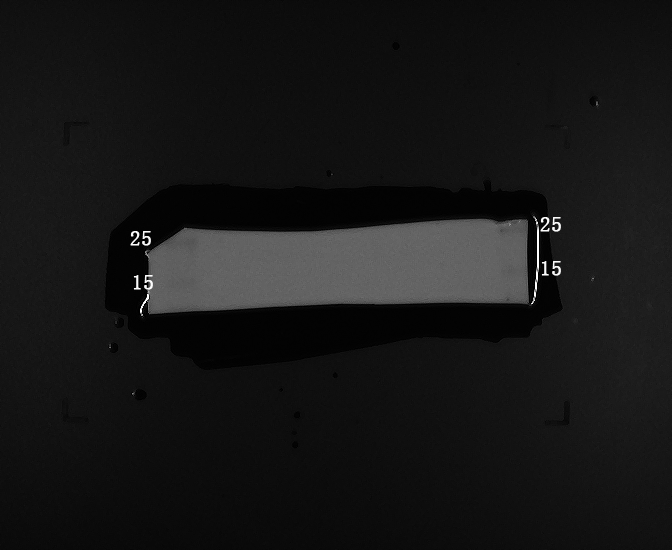
**

**Repeat 3**

**GSDMD 53kDa**

**Repeat 1**

**Repeat 2**

**Repeat 3**

**WB for Supplementary Data**

**β-actin 43kDa**

**Repeat 1**

**Repeat 2**

**Repeat 3**

**NLRP3 118kDa**

**Repeat 1**

**Repeat 2**

**Repeat 3**
